# Sigma 54-Regulated Transcription Is Associated with Membrane Reorganization and Type III Secretion Effectors during Conversion to Infectious Forms of Chlamydia trachomatis

**DOI:** 10.1128/mBio.01725-20

**Published:** 2020-09-08

**Authors:** Katelyn R. Soules, Scott D. LaBrie, Benjamin H. May, P. Scott Hefty

**Affiliations:** a Department of Molecular Biosciences, University of Kansas, Lawrence, Kansas, USA; University of Utah

**Keywords:** *Chlamydia trachomatis*, gene regulation, regulon, sigma factors

## Abstract

*Chlamydia* bacteria are obligate intracellular organisms with a phylum-defining biphasic developmental cycle that is intrinsically linked to its ability to cause disease. The progression of the chlamydial developmental cycle is regulated by the temporal expression of genes predominantly controlled by RNA polymerase sigma (σ) factors. Sigma 54 (σ^54^) is one of three sigma factors encoded by *Chlamydia* for which the role and regulon are unknown. CtcC is part of a two-component signal transduction system that is requisite for σ^54^ transcriptional activation. CtcC activation of σ^54^ requires phosphorylation, which relieves inhibition by the CtcC regulatory domain and enables ATP hydrolysis by the ATPase domain. Prior studies with CtcC homologs in other organisms have shown that expression of the ATPase domain alone can activate σ^54^ transcription. Biochemical analysis of CtcC ATPase domain supported the idea of ATP hydrolysis occurring in the absence of the regulatory domain, as well as the presence of an active-site residue essential for ATPase activity (E242). Using recently developed genetic approaches in *Chlamydia* to induce expression of the CtcC ATPase domain, a transcriptional profile was determined that is expected to reflect the σ^54^ regulon. Computational evaluation revealed that the majority of the differentially expressed genes were preceded by highly conserved σ^54^ promoter elements. Reporter gene analyses using these putative σ^54^ promoters reinforced the accuracy of the model of the proposed regulon. Investigation of the gene products included in this regulon supports the idea that σ^54^ controls expression of genes that are critical for conversion of *Chlamydia* from replicative reticulate bodies into infectious elementary bodies.

## Abstract (precis)

The factors that control the growth and infectious processes for *Chlamydia* are still poorly understood. This study used recently developed genetic tools to determine the regulon for one of the key transcription factors encoded by *Chlamydia*, sigma 54. Surrogate and computational analyses provide additional support for the hypothesis that sigma 54 plays a key role in controlling the expression of many components critical to converting and enabling the infectious capability of *Chlamydia*. These components include those that remodel the membrane for the extracellular environment and incorporation of an arsenal of type III secretion effectors in preparation for infecting new cells.

## INTRODUCTION

Sigma 54 (σ^54^) is a widely distributed and unique subunit of RNA polymerase (RNAP) holoenzyme that is associated with stringent regulation of gene products connected with various critical biological functions in bacteria (1–3). Sigma 54, otherwise referred to as σ^N^, was originally characterized as responding to nitrogen levels in Escherichia coli and *Salmonella* (1, 4). It has since been shown that σ^54^ regulons are diverse and show responses to antibacterial compounds and toxic heavy metals, metabolism of alternative carbon sources, and biosynthesis of pilin and type III secretion systems (T3SS) (1, 5–20). While these signals and responses are variable, transport and biosynthesis of components that comprise the exterior of the bacteria and host cell interactions are themes that appear to be shared among bacterial σ^54^ regulation factors (2).

There are many aspects of σ^54^ transcriptional initiation that are highly conserved between bacterial phyla and distinct from those of σ^70^ family members (21). A primary difference is that the promoter recognized by σ^54^ is centered at the −12 and −24 positions upstream of the transcriptional start site (TSS) rather at than the typical −10 and −35 positions (22, 23). Unlike σ^70^-type sigma factors, which are able to spontaneously separate double-stranded DNA and initiate transcription after forming the RNAP holoenzyme (3, 24), sigma 54 is incapable of transitioning from the closed complex to the open complex on the DNA without the assistance of an ATP-hydrolyzing response regulator (typically referred to as NtrC).

Sigma 54 response regulators are typically composed of three domains: a DNA-enhancer binding domain; an effector ATPase domain with a conserved glutamate-242 residue that polarizes the ATP molecule, enabling hydrolysis to occur; and a receiver domain that is phosphorylated by a sensor kinase (NtrB) in response to an environmental cue (4, 25–27). Phosphorylation of this receiver domain relieves inhibition of the ATPase domain and allows NtrC to hydrolyze ATP, enabling the σ^54^-RNA holoenzyme to form an open DNA complex and initiate transcription (1, 25). Previous studies have shown that in the absence of the receiver domain, the ATPase domain alone can initiate σ^54^-directed transcription (28–30). The regulatory cues that trigger the signaling cascade activating σ^54^ differ in various bacterial species, as do the regulons of genes that σ^54^ is responsible for transcribing. Despite the variations in the activating signals and subsets of genes being regulated in different bacteria, this mechanism of regulation provides tight control of the σ^54^ regulon that is expressed only under specific conditions (1).

*Chlamydia* bacteria are obligate intracellular organisms that code for a σ^54^ homolog, along with two σ^70^ family factors (σ^66^ and σ^28^). σ^66^ and σ^28^ have previously been shown to be important for controlling various stages of temporal gene expression during the developmental cycle of *Chlamydia* (31, 32); however, the role of σ^54^ has yet to be determined. The metabolically active and replicative form of *Chlamydia* is termed the reticulate body (RB), for which σ^66^ directs transcription of most (∼80%) of the encoding genes, and most of the products are associated with metabolism, replication, and maintenance of the intracellular environment (33, 34). As the developmental cycle progresses, RBs asynchronously convert into the infectious, spore-like form of *Chlamydia*, termed the elementary body (EB). During this stage of the developmental cycle, when RB-to-EB conversion is occurring, global gene expression profiling supports the hypothesis that about 20% of the genome is upregulated (33, 34). Expression of many of these genes has been shown to be regulated by σ^66^, with additional factors, such as DNA topology and supercoiling, expected to contribute to this differential expression mechanism (35–37). σ^28^ has been shown to regulate few (but critical) genes during the RB-to-EB conversion process, including those involved in the formation of the condensed DNA structure that is unique to EBs (38–42).

Chlamydial EBs are transcriptionally silent due to a highly condensed nucleoid and have limited metabolic activity and an extensive network of disulfide cross-linked outer membrane proteins for osmotic stability. In spite of the minimal metabolic activity, EBs have extensive infectious capabilities that enable host cell adherence, invasion, and the establishment of the intracellular environment (43). EBs have been shown to bind to host cells using multiple membrane proteins (OmcB, OmpA, and Pmps) and subsequently invade and establish an intracellular vacuolar environment (i.e., inclusion) through the use of a type III secretion system and preformed effector proteins, such as TarP, Incs, and others (44, 45). Because EBs have minimal metabolic activity, the proteins needed for these critical infection processes need to be prepackaged before the RB-to-EB conversion and exit from an infected cell (46). Therefore, at the late stages of the developmental cycle, transcription of genes for membrane remodeling and structural transition to the EB and the arming of necessities for reinfection must occur. While many aspects of the temporal regulation of the developmental cycle have been determined, there are still gaps in the understanding of important details, including how the chlamydial cells regulate the equipping of the EB with the necessary components for survival in the extracellular environment and the next round of host cell infection.

In this study, the regulon of σ^54^ in the human pathogen Chlamydia trachomatis was determined using the ATPase domain of the NtrC homolog and CtcC response regulator protein. Computational investigation of σ^54^ promoter elements and reporter gene expression analyses supported the accuracy of determination of this regulon. From the proposed regulon, σ^54^ is hypothesized to regulate transcription of genes involved in the RB-to-EB transition, including genes encoding many membrane proteins and type III secretion effector proteins known to be important for early stages of EB infection.

## RESULTS

### The effector (ATPase) domain of chlamydial σ^54^ activator protein CtcC exhibited ATP hydrolysis in the absence of a regulatory domain.

**FIG 1:**
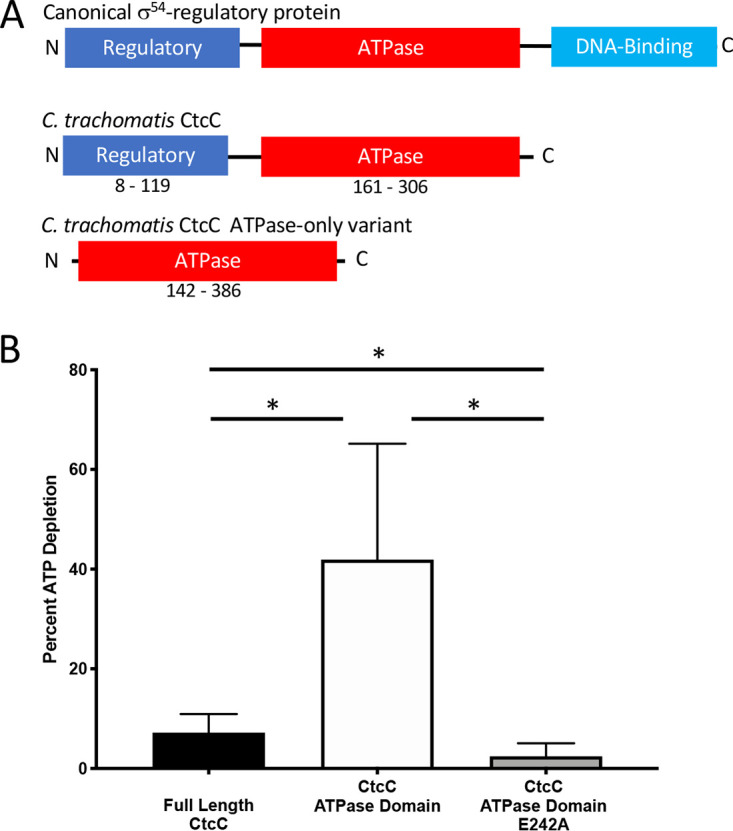
C. trachomatis CtcC domain organization and *in vitro* ATPase activity of CtcC protein constructs. (A) Graphic domain structure of the canonical σ^54^-regulatory protein showing that the native chlamydial homolog, CtcC, does not contain a DNA-binding domain and that the ATPase domain-only variant eliminates the regulatory domain. (B) Triplicate ATPase hydrolysis activity of full-length, ATPase domain, and active-site-defective σ^54^-regulatory protein CtcC. The ATPase domain-only recombinant protein depleted approximately four times as much ATP on average as the full-length CtcC protein. When the E242A substitution was introduced into the ATPase domain, there was a significant decrease in the amount of ATP depletion compared to the full-length CtcC, supporting the hypothesis that this single amino acid substitution disrupts the ability for the protein to perform its normal activity. *, *P* value of <0.05 by Student's *t* test.

CtcC consists of an N-terminal regulatory domain and a C-terminal effector ATPase domain but lacks a DNA-binding domain (Fig. 1A). Figure S1 in the supplemental material shows an amino acid alignment of the chlamydial CtcC protein with homologous response regulatory proteins from other bacterial species. Without the DNA-binding domain, the chlamydial CtcC protein was found to have the highest sequence identity with AtoC from E. coli at 42.2% (62% sequence similarity) (47). Multiple features are conserved between CtcC and other NtrC homologs, including the E242 residue important for ATP hydrolysis (see Fig. S1 in the supplemental material, red arrow), the predicted σ^54^ interaction site (Fig. S1, red box), and the site of phosphorylation by the sensor kinase protein (Fig. S1, red asterisk) (48).

10.1128/mBio.01725-20.1 FIG S1 Clustal W sequence alignment of CtcC from Chlamydia trachomatis (Ct) with other σ^54^-regulatory proteins from Sinorhizobium meliloti (Sm), *Salmonella* Typhimurium (St), Pseudomonas aeruginosa (Pa), and Escherichia coli (Ec) shows that the overall domain structure is conserved among bacterial species. The residues involved in σ^54^ interaction are highly conserved (red box), as is the E242 residue (red arrow) responsible for polarization of the ATP molecule allowing for hydrolysis and the D54 residue that is phosphorylated by the sensor kinase protein. Download FIG S1, PDF file, 0.4 MB. Copyright © 2020 Soules et al. 2020 Soules et al. This content is distributed under the terms of the Creative Commons Attribution 4.0 International license.

The response regulator CtcC (CT466) has previously been shown to be phosphorylated *in vitro* by the sensor kinase CtcB (CT467) (47); however, ATPase activity or the inhibitory effect of the regulatory domain has yet to be investigated. To assess this, *in vitro* ATPase activity was evaluated for full-length CtcC, CtcC with an ATPase domain (amino acids [aa] 142 to 386), and CtcC with an ATPase domain with an active-site substitution (E242A). The full-length CtcC protein exhibited very low ATPase activity, with less than a 10% depletion of total ATP (Fig. 1). In contrast, the ATPase domain reduced ATP levels by over 40%. When an active-site substitution (E242A) was introduced into the ATPase domain, ATPase activity was severely affected. Overall, these data support the idea that the absence of the regulatory domain enables ATPase activity of the ATP domain of CtcC.

### Expression of the ATPase domain revealed the σ^54^ regulon in C. trachomatis.

**FIG 2:**
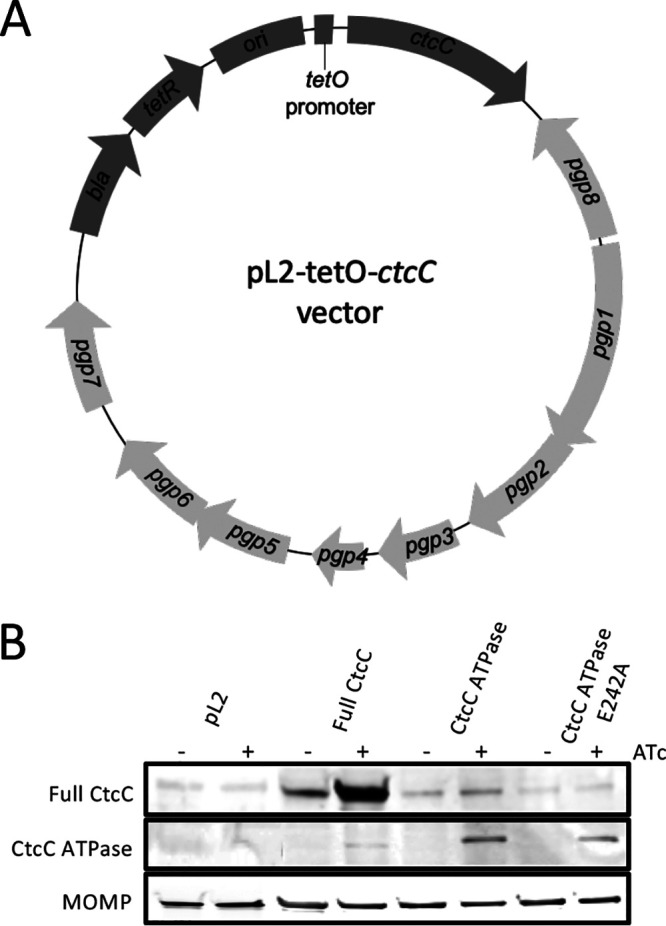
CtcC construct overexpression with inducible vector. (A) Vector map with *ctcC* gene variants under the control of an ATc-inducible promoter on C. trachomatis native plasmid backbone (light gray) (49). (B) Western blot showing overexpression of full-length CtcC, CtcC ATPase domain-only CtcC, and CtcC with an ATPase domain with an E242A substitution induced with addition of ATc after infection at 12 hpi and harvest at 24 hpi.

Previous studies expressing only the ATPase effector domain of NtrC homologs have demonstrated constitutive activation of σ^54^-directed transcription in Salmonella enterica serovar Typhimurium and Sinorhizobium meliloti (28, 30). Given the observations revealing that the CtcC ATPase domain exhibited ATP hydrolysis activity, expression of this domain in *Chlamydia* was hypothesized to activate σ^54^ RNAP and enable the discovery of a cognate regulon. Before evaluating the transcriptional profile, protein induction of full-length CtcC, CtcC with an ATPase domain, or CtcC with an E242A ATPase-inactive variant was evaluated in *Chlamydia* (Fig. 2) (49). Limited but equal levels of expression of wild-type CtcC were detected in vector control samples (Fig. 2B). Full-length CtcC was strongly induced; however, CtcC levels were considerably higher than native expression levels prior to induction, indicating leaky expression (Fig. 2B). With the overexpression of the full-length CtcC protein, there appeared to be a faint band of approximately the same size as the ATPase domain band as well, and while it is possible that this might have represented a degradation product, the identity of this band has not been fully investigated. Importantly, induced expression of the ATPase domain and of the E242A variant was evident (Fig. 2B).

To discover a potential σ^54^ regulon in *Chlamydia*, RNA was isolated from infected samples with vector only or from a construct harboring the ATPase domain after 4 h of induction (16 to 20 h postinfection [hpi]). Stranded RNA sequencing (RNA-seq) analysis was performed on RNA from two biological replicates, with results revealing that transcripts for 64 genes were significantly and reproducibly upregulated in both samples following induced expression of the CtcC ATPase domain (Table 1; see also Table 2). An initial and striking observation related to this potential regulon was that all of the genes found to be upregulated following ATPase domain induction are normally upregulated during the middle-late stages of the *Chlamydia* developmental cycle (33, 34). These stages coincide with the RB-to-EB conversion, as well as with other late infection and exit events. Twenty-four genes are classified as mid-late-stage genes and are normally upregulated after 16 to18 hpi during the developmental cycle, and 40 genes are late-stage genes, normally upregulated after 24 hpi (33, 34). Of the 64 genes, 41 (64%) were found to encode hypothetical proteins with no known or only putative functions, and 23 (36%) were functionally annotated. Nineteen of the genes code for membrane proteins, 6 of which are predicted to be inclusion membrane proteins. Twenty-eight genes encoded type 3 secretion system (T3SS)-associated proteins, including effector proteins such as TarP.

**TABLE 1:** Genes directly regulated by σ^54^

CT no.^*a*^	Locus tag	Gene name	Protein role	Reference(s)	RNA-seq avg differential expression (fold)	Distance from start codon (nt) or category
Mid-late genes						
CT051	CTL0307	hyp	Putative secreted, Pmp-like protein	81	6.58	67
CT105^*d*^	CTL0360	hyp	Hypothetical	100	3.12	178^*c*^
CT142	CTL0397	hyp	T3SS exported protein	84–85	4.81	95
CT143	CTL0398	hyp	T3SS exported protein	84–85	4.79	Operon
CT144	CTL0399	hyp	T3SS exported membrane protein	84–85	3.99	Operon
CT455^*d*^	CTL0715	*murA*	T3SS secreted protein; UDP-N-acetylglucosamine 1-carboxyvinyltransferase	57	3.01	16
CT494	CTL0755	*sohB*	Exported protease	100	5.02	105
CT575	CTL0838	*mutL*	DNA mismatch repair protein	100	4.76	56
CT635	CTL0003	hyp	Putative T3SS chaperone	87	5.48	248
CT683	CTL0052	hyp	Tetratricopeptide repeat protein	100	3.59	40
CT849.1	CTL0222	hyp	Hypothetical	100	4.18	43

Late genes						
CT005	CTL0260	inc	Inclusion membrane protein	77–79	3.61	42
CT050	CTL0306	hyp	Secreted, Pmp-like protein	81	6.1	186
CT082	CTL0338	hyp	Putative T3SS effector	82	3.4	116
CT083	CTL0338A	hyp	Putative T3SS secreted protein; putative thymidylate kinase	88	3.96	Operon
CT084	CTL0339	PLD	Phosphatidylcholine-hydrolyzing phospholipase D (PLD) protein	96	4.68	176
CT229^*b*^	CTL0481	inc	Inclusion membrane protein	66–67	3.67	37
CT394	CTL0650	hrcA	Heat-inducible transcriptional repressor	73–74	4.5	18
CT395	CTL0651	*grpE*	HSP-70 cofactor	89	3.59	Operon
CT443^*d*^	CTL0702	*omcB*	Membrane protein	55	3.09	Operon
CT444	CTL0703	*omcA*	Membrane protein	55	4.6	165
CT489	CTL0750	*glgC*	Glucose-1-phosphate adenylyltransferase	100	4.79	136
CT493	CTL0754	*polA*	DNA polymerase A	90	5.03	16
CT619	CTL0883	hyp	Putative T3SS effector	91–92	5.66	36^*c*^
CT620	CTL0884	hyp	Putative T3SS effector	86, 92	6.48	198
CT622	CTL0886	hyp	T3SS effector; putative cell surface protein	87	3.51	42
CT702	CTL0071	hyp	Hypothetical	100	4.29	2
CT711	CTL0080	hyp	Putative T3SS effector	86, 92	4.36	83^*c*^
CT814	CTL0185	hyp	Putative membrane protein	100	3.55	66
CT814.1	CTL0186	hyp	Putative membrane protein	100	5.24	102
CT841^*d*^	CTL0213	*ftsH*	Membrane reorganization protease	100	3.1	28
CT847	CTL0219	hyp	Putative T3SS effector	93	4.03	133
CT875	CTL0255	hyp	T3SS effector	94	3.83	180^*c*^

a Expression pattern classifications were taken from previous microarray studies (33, 34). Late-stage genes are upregulated at 24 hpi; mid-late genes are upregulated at 18 hpi.

b Expression pattern evaluated for differentially regulated transcript levels evaluated by ddPCR (Table S2).

c Transcript reads from RNA-seq data suggest a different transcriptional start site compared to current C. trachomatis 434/Bu genome (NC010287). Figure S3 shows PCR confirmation of alternative transcriptional start site.

d Two standard deviations from average in one of two RNA-seq experiments.

**TABLE 2:** Genes indirectly regulated by σ^54^

CT no.	Locus tag	Gene name	Protein role	Reference(s)	RNA-seq avg differential expression (fold)
Mid-late genes^*a*^					
CT049	CTL0305	hyp	Secreted, Pmp-like protein	81	4.63
CT132	CTL0387	hyp	Putative membrane protein	100	3.89
CT157^*c*^	CTL0413	PLD	T3SS secreted protein; phospholipase D	82, 96	4.16
CT196^*c*^	CTL0448	hyp	Putative inclusion membrane protein	95	3.11
CT218	CTL0470	*surE*	5-Nucleotidase	100	3.49
CT382.1^*c*^	CTL0638	hyp	Hypothetical	100	3.09
CT547^*c*^	CTL0809	hyp	Putative exported protein	100	2.91
CT646^*b*^	CTL0014	hyp	Hypothetical	100	4.32
CT647	CTL0015	hyp	Putative exported protein (lipoprotein)	100	4.84
CT648	CTL0016	hyp	Putative membrane protein	100	3.90
CT798	CTL0167	*glgA*	Glycogen synthase; secreted protein	97	4.14
CT837	CTL0209	hyp	Putative inclusion membrane protein	98	4.54
CT849^*c*^	CTL0221	hyp	T3SS secreted protein	84	3.56

Late genes^*a*^					
CT046	CTL0302	*hctB*	Histone-like protein	39–41, 80	3.31
CT073	CTL0329	hyp	Putative outer membrane protein	100	3.18
CT080	CTL0335	*ltuB*	Late transcription unit B protein	70	5.23
CT181^*c*^	CTL0433	hyp	Putative exported protein	100	3.29
CT288	CTL0540	hyp	Putative inclusion membrane protein	83	5.73
CT356	CTL0610	hyp	Thioredoxin domain-containing protein	100	4.15
CT365^*c*^	CTL0619	hyp	Putative inclusion membrane protein	94	2.98
CT392^*c*^	CTL0648	hyp	Hypothetical	100	3.39
CT456	CTL0716	*tarp*	Translocated actin-recruiting phosphoprotein	44, 64	4.75
CT546	CTL0808	hyp	Putative outer membrane protein	100	3.35
CT565^*c*^	CTL0828	hyp	Putative membrane protein	78	3.36
CT576	CTL0839	*scc2*	T3SS chaperone	37	4.83
CT577	CTL0840	hyp	Putative cytosolic protein	37	5.10
CT578	CTL0841	*copB*	Putative T3SS membrane protein	37	4.53
CT579	CTL0842	*copD*	Putative T3SS protein	37	3.93
CT682^*c*^	CTL0051	*phpB*	Penicillin-binding protein	56	2.97
CT694	CTL0063	hyp	Putative T3SS effector	99	3.39
CT848	CTL0220	hyp	T3SS secreted protein	88	3.72

a Expression pattern classifications were taken from previous microarray studies (33, 34). Late-stage genes are upregulated at 24 hpi; mid-late genes are upregulated at18 hpi.

b Expression pattern evaluated for differentially regulated transcript levels evaluated by ddPCR (Table S2).

c Two standard deviations from average in one of the two RNA-seq experiments.

To validate this expression profile, droplet digital PCR (ddPCR) was performed on newly isolated RNA for a subset of 19 genes selected from the σ^54^ overactivation RNA-seq data (Table 1; see also Table 2). All of these genes displayed upregulation following induction of the CtcC ATPase domain (see Table S1 in the supplemental material). Several genes evaluated by ddPCR were found to have expression ratios more than twice as high as those seen with RNA-seq. This difference is possibly due to the additional processing that RNA prepared for RNA-seq must go through, including exonuclease treatment and rRNA depletion steps, although RNA stability and loss have not been evaluated for each step (50). To determine if pleiotropic transcription effects should be considered, the expression ratio was determined for *rpoA*, which encodes the alpha subunit of RNA polymerase and is transcribed by σ^66^ holoenzyme. Expression levels of *rpoA* were unchanged following induction of the CtcC ATPase domain. To demonstrate that transcription induction was specific to CtcC ATPase domain activity, ratios were also determined following induction of the E242A ATPase domain. Levels of transcription of the 19 σ^54^ regulon genes and *rpoA* following induction of the ATPase-defective form were unchanged relative to the vector control sample (Table S1).

10.1128/mBio.01725-20.7 TABLE S1 Average differential expression ratio (ATPase versus vector control). Download Table S1, PDF file, 0.02 MB. Copyright © 2020 Soules et al. 2020 Soules et al. This content is distributed under the terms of the Creative Commons Attribution 4.0 International license.

10.1128/mBio.01725-20.8 TABLE S2 Temporal gene expression level evaluation of *ct229* and *ct646*. Download Table S2, PDF file, 0.05 MB. Copyright © 2020 Soules et al. 2020 Soules et al. This content is distributed under the terms of the Creative Commons Attribution 4.0 International license.

When the full-length CtcC construct was induced, transcript ratios for most of the 19 genes were found to be considerably lower than those observed with the ATPase domain, supporting the idea that the regulatory domain was still inhibiting ATPase activity *in vivo*, similarly to the observations of the *in vitro* ATPase depletion study (Fig. 1). Interestingly, many σ^54^ genes (*ct456*, *ct619*, *ct620*, *ct683*, *ct711*, and *ct875*) were transcribed at ratios twice those observed for the ATPase-defective domain (E242A) whereas *rpoA* levels were unchanged. This observation suggests that overexpression of the full-length CtcC enabled transcriptional activation for some σ^54^ genes. This limited transcriptional activation might have been contributed by the minimal ATPase activity, as was observed *in vitro* for the full-length form (Fig. 1B), but amplified by protein overexpression. Alternatively, it might have been due to CtcC autophosphorylation or CtcB phosphorylation of expressed CtcC and subsequent transcriptional activity.

Overall, RNA-seq and ddPCR validation studies support the identification of a putative σ^54^ regulon in C. trachomatis that is associated with mid-late-stage gene expression and RB-to-EB conversion components. To confirm that these components were expressed and present during these stages of the developmental cycle, ddPCR was used to calculate the transcript levels of *rpoN* (σ^54^), *ctcB*, and *ctcC* at 12, 18, 24, and 30 hpi. The transcript levels for σ^54^ were found to be relatively constant, while *ctcC* and *ctcB* levels increased throughout the developmental cycle (Fig. S2). Interestingly, the CtcB sensor kinase had very low levels of transcription early in the developmental cycle before they rose above those of σ^54^ after 24 hpi. These results mirror those in prior studies in support of the timing and function of this gene regulatory system (33, 34, 51).

10.1128/mBio.01725-20.2 FIG S2 Temporal analysis of *ctcC, ctcB*, and *rpoN* transcript levels throughout the chlamydial developmental cycle. RNA was isolated at 12, 18, 24, and 30 hpi from a wild-type L2 infection. Transcript counts were determined by ddPCR and normalized to *secY* transcript counts. *rpoA* was used as a constitutively active control. Download FIG S2, PDF file, 0.04 MB. Copyright © 2020 Soules et al. 2020 Soules et al. This content is distributed under the terms of the Creative Commons Attribution 4.0 International license.

10.1128/mBio.01725-20.3 FIG S3 Alignment of σ^54^ promoters upstream of differentially regulated genes. Predicted σ^54^ promoters for each gene are shown along with the computational promoter prediction scores (indicated in parentheses). These alignments show that the canonical TGG-N_9_-TGC σ^54^ promoter sequence is conserved in these predicted promoter regions. Download FIG S3, PDF file, 0.1 MB. Copyright © 2020 Soules et al. 2020 Soules et al. This content is distributed under the terms of the Creative Commons Attribution 4.0 International license.

### Computational analysis identified σ^54^ promoter sequences upstream of 33 σ^54^ regulon genes.

**FIG 3:**
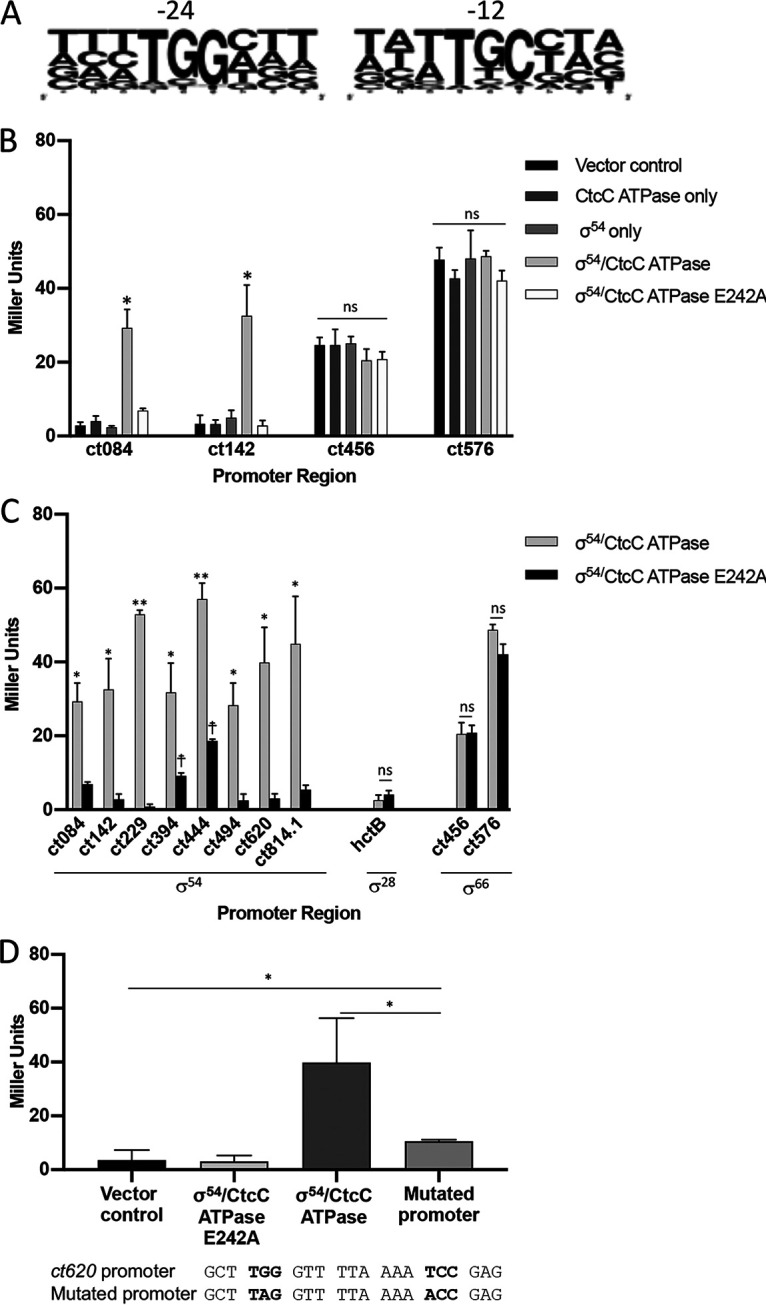
Beta-galactosidase assays of σ^54^- and CtcC-dependent activation on selected promoters. Promoter regions upstream of selected σ^54^-regulated genes were tested for their ability to induce expression of *lacZ* on the pACYC vector with or without the chlamydial *rpoN* gene (*σ^54^*) and *ctcC ATPase* gene (pRSF-DUET). (A) WebLogo alignment of the −12/−24 promoter elements shows the relative frequencies of nucleotides of the predicted σ^54^ promoter regions upstream of the genes that were found by RNA-seq to be differentially regulated. IFU, inclusion-forming units. (B) Promoters were used to test the induction of *lacZ* expression without either σ^54^ or CtcC present (vector control) and with each protein expressed individually in order to assess the system for induction from the E. coli σ^54^. (C) Additional promoters were tested from the predicted σ^54^ regulon, looking at the difference between the constitutively active CtcC ATPase and the inactivated E242A mutant. The predicted regulatory sigma factor is indicated below the promoter region. Experiments were performed in triplicate. * and **, *P* values of <0.05 and <0.01, respectively, by Student's *t* test comparing the CtcC ATPase to the E242A ATPase mutant; †, *P* value of <0.05 by Student's *t* test comparing the CtcC E242A ATPase mutant to a promoterless pACYC-*lacZ* negative control. ns, not significant. (D) Two point mutations were introduced into the *ct620* promoter (alignment below graph). The level of LacZ activity was decreased significantly compared to the wild-type promoter but was still increased significantly compared to the inactive conditions. *, *P* value of <0.05 by Student's *t* test. All beta-galactosidase assays were performed in triplicate.

One study has investigated the presence of σ^54^ promoters in *Chlamydia*, with results indicating that nine genes are preceded by a consensus sequence (52). Given that 64 genes appear to be part of the σ^54^ regulon, a broader computational investigation of putative σ^54^ promoters in the C. trachomatis genome was applied. The σ^54^ consensus sequence TGG-N_9_-TGC, allowing one mismatch and a variable length of linker region of between 8 and 10 nucleotides (nt), within 400 bases upstream of a putative σ^54^ gene target was used for the search (23). Of the 64 putative σ^54^-regulated genes, 28 were found to be preceded by a σ^54^ promoter consensus sequence and are considered to be direct σ^54^ targets (Table 1; see also Fig. S3). Five genes that were included in the σ^54^ regulon were not preceded by σ^54^ promoters but are expected to be contained within an operon that is controlled by σ^54^ promoter (Table 1; operon). These 28 promoters were used to generate a consensus *Chlamydia* σ^54^ promoter that reflects the diversity of nucleotides at individual sites (WebLogo; Fig. 3A) (53).

Interestingly, four genes (*ct105*, *ct619*, *ct711*, and *ct875*; all hypothetical proteins) have predicted σ^54^ promoters inside the currently annotated open reading frame; however, based on the RNA-seq transcript reads, the transcriptional start site (TSS) appears to be downstream from the start codon and is in line with the σ^54^ promoter (Fig. S4). In order to investigate if the transcriptional start sites are internal components of the annotated start codon and to validate the σ^54^ promoter for these genes, primers were designed to lie on either side of the alignment of the RNA-seq reads (Table S4). PCR of cDNA confirmed that transcripts occurred only with the internal primers for all four genes, indicating that the transcripts start internally to the annotated start codon for these genes. A new start codon for these genes was selected as the next in-frame ATG, and the location of that start codon is indicated as the distance from the σ^54^ promoter (Table 1). These observations support the idea that 33 genes are direct targets of σ^54^ (Table 1). This also suggests that 31 genes are indirect σ^54^ targets and are regulated by an unknown factor(s) (Table 2).

10.1128/mBio.01725-20.4 FIG S4 PCR analysis of transcriptional start site comparing the current annotated open reading frame to the transcript read alignments observed by RNA-seq for four hypothetical proteins with predicted σ^54^ start sites that would affect the open reading frame of the transcribed gene. Primers were designed to sit just on either side of the transcript read alignment (blue arrow below gene), with primer 1 amplifying a region that would capture transcripts for the annotated open reading frame (ORF) and primer 2 amplifying a region internal of the transcript alignments. The same internal reverse primer was used for PCR amplification with both primer 1 and primer 2. The approximate location of the next in-frame start codon downstream from the σ^54^ promoter is represented by the red box. Above each schematic is the DNA sequence corresponding to the region that included the σ^54^ promoter (highlighted in yellow), the originally annotated start codon (bold), and the next in-frame ATG downstream of the σ^54^ promoter (red) with a possible ribosomal binding site within a reasonable distance (underlined, if present). To the right are gel images showing PCR products for genomic DNA or cDNA made from isolated chlamydial RNA for each primer set. With these four genes encoding hypothetical proteins, they show amplification only with the primer internal to the RNA-seq transcript alignment, supporting the idea that the transcriptional start site is downstream from the currently annotated start codon for the gene. Download FIG S4, PDF file, 0.2 MB. Copyright © 2020 Soules et al. 2020 Soules et al. This content is distributed under the terms of the Creative Commons Attribution 4.0 International license.

### Reporter gene assays supported σ^54^- and ATPase-dependent regulation of selected σ^54^ gene promoters.

To provide further support for direct targets of σ^54^-mediated transcription, β-galactosidase assays were performed in E. coli with inducible *Chlamydia* σ^54^ and CtcC ATPase domain. Induction of *Chlamydia* σ^54^ and CtcC ATPase proteins resulted in β-galactosidase activity when *ct084* and *ct142* promoter regions preceded *lacZ* (Fig. 3B). Both of these genes were upregulated with σ^54^ activation and predicted to have σ^54^ promoters (Table 1; see also Fig. S3). σ^54^ or the inactive CtcC ATPase domain, alone or in combination, was unable to initiate transcription (Fig. 3B), supporting the hypothesis of specificity and functionality of this surrogate system. Promoter regions for two genes, *ct456* and *ct576*, did not enable transcription with or without σ^54^ and/or the CtcC ATPase present. These genes were not predicted to have σ^54^ promoters but were upregulated with σ^54^ activation. Interestingly, both *ct456* and *ct576* promoters result in β-galactosidase activity without additional factors, supporting the idea that these promoters are active in E. coli, which corresponds with results of a prior study (37).

Six additional putative σ^54^ promoters were selected for further investigation using this surrogate system and displayed CtcC ATPase-dependent activation (Fig. 3C). The *ct456* and *ct576* promoters again displayed CtcC ATPase-independent transcriptional activity. Transcription of *hctB* was upregulated by the CtcC ATPase domain as shown by both RNA-seq and ddPCR (Table 2; see also Table S1). This gene has been shown to be under σ^28^ regulation in late stages of the chlamydial developmental cycle (39–41, 54) and lacks a σ^54^ promoter. The *hctB* promoter region did not exhibit *lacZ* activation in the presence or absence of σ^54^ (Fig. 3C); however, when the gene encoding σ^28^ (*fliA*) was added in conjunction, there was a significant increase in LacZ activity (Fig. S5), showing that the cloned-in promoter region is functional.

10.1128/mBio.01725-20.5 FIG S5 Beta-galactosidase assays for additional investigation of upstream regions of *hctB.* The *hctB* upstream region does not show activation of the *lacZ* gene with expression of σ^54^ and CtcC; however, when σ^28^ is present, LacZ activation is detected. *, *P* value of <0.05 (by Student’s *t* test). Download FIG S5, PDF file, 0.05 MB. Copyright © 2020 Soules et al. 2020 Soules et al. This content is distributed under the terms of the Creative Commons Attribution 4.0 International license.

The upstream regions for *ct394* and *ct444* showed a significant increase in β-galactosidase activity with the CtcC ATPase E242A mutant compared to the promoterless pACYC-*lacZ* control (Fig. 3C). While the level of β-galactosidase activity seen with CtcC ATPase was significantly greater than that seen with inactive protein, a closer analysis of the upstream regions for *ct394* and *ct444* revealed that both σ^54^ and σ^66^ promoter sequences were present for these genes (Fig. S6). Interestingly, the σ^54^ promoter for *ct394* lies inside the σ^66^ promoter, allowing the possibility that σ^54^ would act as an inhibitor of transcription for this gene when no CtcC activation occurred. For both genes, the presence of these dual promoters could account for the low level of β-galactosidase activity seen when no σ^54^/CtcC ATPase proteins were present.

10.1128/mBio.01725-20.6 FIG S6 Upstream regions for *ct394* and *ct444* show both σ^54^ and σ^66^ promoter regions present. Promoter sequences are highlighted in yellow with the downstream start codon for the gene underlined. Interestingly, the σ^54^ promoter lies inside the σ^66^ promoter for *ct394*. Download FIG S6, PDF file, 0.05 MB. Copyright © 2020 Soules et al. 2020 Soules et al. This content is distributed under the terms of the Creative Commons Attribution 4.0 International license.

To further validate the σ^54^ consensus sequence, two conserved bases in the *ct620* promoter were mutated and β-galactosidase activity evaluated. The central G at position −24 and the T at position −13 were both mutated to A. The level of β-galactosidase activity promoted by this mutated sequence was significantly lower than that seen with *lacZ* driven by the wild-type promoter. The β-galactosidase levels for the mutated promoter were still higher than those seen with the vector control and with the E242A CtcC ATPase mutant (*P* value = 0.0339) (Fig. 3D), supporting the idea that the promoter was still functional. These data suggest that the predicted promoter sequence is necessary for σ^54^-initiated transcription.

### Overexpression of CtcC protein variants in *Chlamydia* resulted in an abnormal phenotype and lower levels of progeny production.

**FIG 4:**
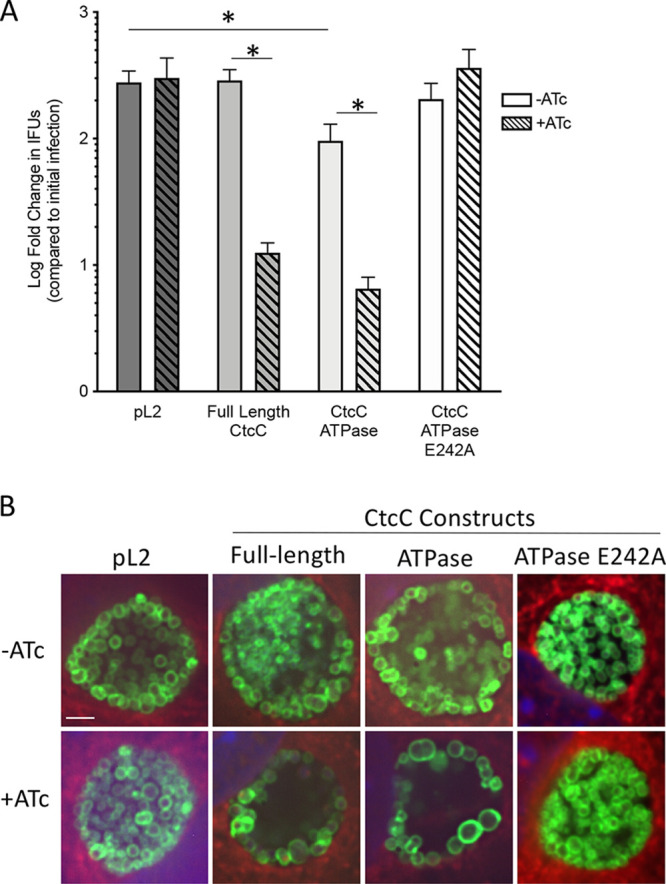
Phenotypic analysis of CtcC variant overexpression during host cell infection. (A) Progeny assay showing a significant decrease in progeny production with overexpression of the full-length and ATPase-only CtcC protein variants. The vector control (pL2) showed no difference in progeny production with the addition of ATc. Induction of full-length CtcC at 12 hpi resulted in a decrease in the number of progeny passaged at 30 hpi compared to the results seen with the pL2 control and the uninduced conditions. Overexpression of the CtcC ATPase domain-only construct resulted in less progeny as well. Additionally, the uninduced CtcC ATPase domain-only infection resulted in a significant difference in progeny produced compared to the pL2 vector control. No difference in progeny production was observed with the E242A substitution in the ATPase domain. *, *P* value of <0.05. (B) Immunofluorescent microscopy of C. trachomatis-infected cells following expression induction of CtcC variants. Induction of full-length CtcC appears to have affected the observable chlamydial cell density inside the center of the inclusion. Overexpression of the ATPase domain-only variant caused the chlamydial cells to have an enlarged appearance, in addition to having fewer cells apparent in center of the inclusion. Infections were induced with 10 ng/ml of ATc at 12 hpi, and the infected cell monolayers were fixed and stained at 24 hpi. Green, OmpA; red, host cytosol; blue, DAPI—DNA. Representative images show at least 5 inclusions each; each image represents an average of 3 projections of z-stack images. Scale bar = 2 μm.

Progeny production was evaluated with *Chlamydia*-infected L929 cells at 30 hpi either with or without 18 h of protein induction. Addition of anhydrotetracycline (ATc) alone had no effect on progeny formation; nor did the induction of the inactive ATPase domain (Fig. 4A). Full-length CtcC induction resulted in a 1.4-log-fold decrease in progeny production, while the ATPase domain caused a 1.6-log-fold-lower progeny production level. When no ATc was added to the pL2-*ctcC* ATPase-only construct chlamydial strain, there was also a significant, 0.5-log-fold decrease in progeny compared to the vector control strain. This decrease was likely due to minimal uninduced production of the CtcC ATPase domain-only protein.

In order to evaluate bacterial cellular morphology and inclusion composition, immunofluorescent microscopy was performed on infected L929 cells with or without 12 h of induction (12 or 24 hpi). Similarly to the progeny production results (Fig. 4A), no observable abnormal bacterial or inclusion morphology was apparent in vector control or ATPase-inactive samples (Fig. 4B). Overexpressing the full-length CtcC protein or ATPase domain resulted in a noticeable decrease in the level of C. trachomatis bacterial cells within the interior of the inclusion, as most organisms were observed almost exclusively around the inclusion membrane (Fig. 4B). Furthermore, the bacterial cells expressing the ATPase domain appeared to be larger, with more diversely sized reticulate bodies. The reduction in the levels of observable organisms in the CtcC full-length and ATPase domain-expressed inclusions (Fig. 4B) matched the reduction of progeny production (Fig. 4A). Importantly, the shared phenotypes observed in CtcC full-length and ATPase domain expression samples are likely a result of pleiotropic effects associated with disrupted signaling and irregular gene expression. Transcriptional analysis of these two samples showed that there was some overlap in gene expression profiles but that the overlap was fairly minimal (Table S1). This could indicate that the induced expression levels of these few shared gene products represent the primary contributors to the disrupted growth phenotypes observed. Finally, the absence of the growth effect with the E242A ATPase-defective mutant supports the idea that ATPase activity and σ^54^ gene expression were the likely causes of these defects, rather than general overexpression of the protein.

## DISCUSSION

In this study, the σ^54^ regulon has been determined, and the results support the idea of a role in controlling RB-to-EB conversion processes, especially those involving many components associated with infectious capabilities. As observed with other NtrC homologs, the absence of the CtcC regulatory domain enabled ATPase activity (Fig. 1B). This activity appears to have enabled the induction of σ^54^ transcription in the absence of CtcC phosphorylation by CtcB. This is evidenced by RNA-seq profiling results (Table 1; see also Table 2), the presence of σ^54^ promoters (see Fig. S3 in the supplemental material), and surrogate reporter gene analyses (Fig. 3). A major observation was that all genes within the resulting σ^54^ regulon were upregulated during the middle and late stages of the developmental cycle, supporting the idea that σ^54^ is critical for controlling gene expression during these developmental phases. Investigation of the products encoded by genes in the σ^54^ regulon further supports the idea of a role in preparing EBs for the next round of infection through remodeling the outer membrane and packaging numerous type III secretion effector proteins. Overall, these observations provide comprehensive support for the previously unknown and critical role of σ^54^ in *Chlamydia*.

All of the genes making up the chlamydial σ^54^ regulon were normally upregulated during mid-late (16 to 18 hpi) or late (24 hpi) stages of the developmental cycle. Interestingly, *omcA* and *omcB* were both found among the genes with σ^54^ promoters and appear to be direct σ^54^ targets. OmcA and OmcB are cysteine-rich proteins that make up a large portion of the highly cross-linked EB cellular envelope whose genes are translated late in the developmental cycle (55). Additionally found to be part of the σ^54^ regulon, *ct682* (*pdpB*), *ct455* (*murA*), and *ct841* (*ftsH*) are all cell wall modification proteins transcribed after 18 hpi (56–58). This collection of late-stage genes associated with cell membrane composition suggests that the σ^54^ regulon is involved in the RB-to-EB transition. EBs are highly condensed forms of the chlamydial cell with a large degree of disulfide bonds holding the membrane rigid against osmotic pressure outside the host cell, compared to the fragile membrane of RBs (59–61). Thus, in late stages of the developmental cycle, the transcription of genes involved in this membrane recomposing and restructuring is critical for the transition between chlamydial forms.

Just under half of the σ^54^ regulon contains genes encoding T3SS-associated proteins, including secreted effectors, inclusion membrane proteins, parts of the T3SS apparatus, and chaperone proteins. The T3SS is essential for chlamydial virulence and initially establishing an infection in a host cell (62, 63). TARP (for translocated actin recruiting phosphoprotein) associates directly with actin in the host cell at the site of EB invasion and enhances EB uptake (44, 64, 65). CopB and CopD have been shown to make up the translocator of the T3SS apparatus and are thus essential for attachment of the chlamydial cell to the host membrane (37, 66). CT229 has been shown to be an inclusion membrane protein that is critical for the subverting of the host cell defenses that would otherwise lead to destruction and therefore is necessary for the establishment of the infection from the initial point of entry (67, 68). These are just a few examples of T3SS-associated genes that were found to be regulated by σ^54^. Because the EBs are less metabolically active than RBs, the many effector proteins needed for establishing the chlamydial inclusion and for commandeering the host cell machinery must be made before the conversion event. With a substantial number of the genes in the σ^54^ regulon coding for T3SS-associated proteins, the data suggest that σ^54^ gene regulation is involved in the preparation of the chlamydial cells for propagation of the infection (46).

The σ^54^ regulon is split almost equally between direct and indirect gene targets (Table 1; see also Table 2). This supports the idea that other regulators participate in controlling the σ^54^ regulon. Currently, only a few transcription factors have been identified for *Chlamydia* (69, 70). More than half (64%) of the genes in the σ^54^ regulon code for hypothetical proteins, several of which have no predicted role or identifiable motifs. Thus, there is a possibility that there is an unknown transcriptional regulator among the genes transcribed by σ^54^ that may contribute to regulating the indirect targets in the regulon. It was interesting to observe that the well-characterized σ^28^ gene target *hctB* was reproducibly and specifically upregulated upon induction of the intact CtcC ATPase domain (Table 2; see also Table S1) (41, 71). The level of upregulation observed here (∼3-fold) is minimal relative to the upregulation observed during temporal expression between 18 and 24 hpi (∼60-fold [37]). The absence of a σ^54^ promoter element (Fig. S3) and lack of induction in E. coli (Fig. 3; see also Fig. S5) support the idea that it is an indirect target of σ^54^. However, upregulation of this DNA condensation factor could have broader effects on gene regulation due to modified DNA topology and supercoiling, an aspect also well established in *Chlamydia* and bacterial gene regulation (72, 73).

Tandem promoters for both σ^54^ and σ^66^ were found to be present upstream of *ct394* (*hrcA*) and *ct444* (*omcA*) (Fig. S6), potentially allowing for different temporal regulation patterns (38). While *hcrA* has been shown to have a σ^66^ promoter *in vitro* and in the E. coli surrogate system (Fig. 3C) (74, 75) and a CIRCE operator element, the presence of the σ^54^ promoter directly inside the −10/−35 sequence might disrupt the ability of σ^66^ to bind to the DNA and thus additionally inhibit transcription of the downstream gene when σ^54^-mediated transcription is inactive. These two genes are just a couple of examples of the potential for multiple layers of regulation that could be at play for any number of genes in the chlamydial genome.

The σ^54^ regulon in *Chlamydia* has previously been investigated only in an *in silico* promoter mapping study (52). Mathews and Timms predicted only nine genes as being regulated by σ^54^ based on the presence of the canonical σ^54^ consensus sequence. In the current study, a less strict consensus sequence (TGG-N_9_-TGC) was used to look for potential σ^54^ promoters. Two of the nine genes (*ct620* and *ct683*) previously predicted by Mathews and Timms to be regulated by σ^54^ appeared in the RNA-seq analysis as being differentially expressed when σ^54^ was overactivated. The potential for additional regulatory factors affecting the transcription of the σ^54^-regulated gene may account for the discrepancy between those genes that were computationally predicted to have σ^54^ promoter sequences and those that were found to be experimentally upregulated by σ^54^ overactivation. For instance, CT398 (CdsZ) was previously shown to interact with σ^54^ by Barta et al. (76) and is hypothesized to be a repressor of σ^54^ activity, although its impact on transcription has yet to be evaluated (76).

While this study addressed the general role of σ^54^ in *Chlamydia*, there are many issues remaining regarding σ^54^ activation. The importance of the timing of σ^54^ activation for the coordinated progression of the developmental cycle is demonstrated by the reduction in infectious progeny produced and the altered morphology of the *Chlamydia* cells with the overexpression of the active CtcC proteins. However, the importance of the synchronization and interdependence of all three of the chlamydial sigma factors has yet to be fully explored, but they are likely to be crucial for the transitions between RB and EB forms.

The lack of an effect on growth seen with the E242A ATPase-defective mutant supports the idea that ATPase activity and σ^54^ gene expression are the likely causes of defects, rather than the general overexpression of the protein (Fig. 4). The limited change in gene expression seen with the E242A ATPase mutant (see Table S1 in the supplemental material) also supports the idea that this protein variant was not having an effect on σ^54^ activation. The data thus suggest that the native CtcC protein does not interact with the E242A ATPase domain-only protein variant, as opposed to having a dominant-negative effect on the native protein activity. There is a chance that the amino acid substitution causes this version of CtcC to be misfolded although this has not been evaluated. Despite this possibility of misfolding, the data still suggest that overexpression of the protein is not solely the cause of the gene expression and phenotypic changes but rather that the changes are a consequence of the activity of the functional CtcC protein.

The signal that initiates the two-component regulatory cascade starting with the sensor kinase CtcB, stimulating CtcC, and subsequently activating σ^54^ in *Chlamydia* has yet to be determined. In bacteria such as *Salmonella* Typhimurium, NtrC becomes phosphorylated by NtrB under nitrogen-limiting conditions (1). The homologous regulatory protein in *Rhizobium*, DctD, is phosphorylated by DctB in response to the availability of external dicarboxylates (29, 30). DctB in Sinorhizobium meliloti and Vibrio cholerae binds to the C_4_-dicarboxylate succinate to signal for the expression of succinate transport proteins (29, 30). In E. coli, the sensor kinase AtoS responds to acetoacetate, leading to the transcription of genes encoding enzymes involved in short-chain fatty acid metabolism (77). While homologs of the sensor kinase and response regulators in similar two-component regulatory systems have been identified in various bacteria, the particular environmental signal in many cases has yet to be identified. Supporting the idea of the incorporation of dicarboxylates and metabolic intermediates as a sensor molecule in *Chlamydia*, recent studies have shown that RsbU binds to tricarboxylic acid (TCA) intermediates to potentially control the main σ factor (σ^66^) (78). Determining the initial signal sensed by CtcB would provide critical information concerning what *Chlamydia* specifically responds to in controlling the developmental cycle and a more extensively defined role of σ^54^. Prior studies have shown that CtcB phosphorylates CtcC *in vitro* (47); however, direct dependence on phosphorylation for ATPase activity or transcriptional activation has not been assessed. Future studies demonstrating that CtcC phosphorylation enables ATPase activity and transcription are important to experimentally complete the signal Ctc transduction model in *Chlamydia*. The σ^54^ gene targets and observations reported here provide a sound basis for these future studies.

A 2011 study published by Francke et al. looked for a common theme in σ^54^ regulons across bacterial phyla (2). Their comparative analyses found that genes associated with flagellar and cellular membrane components in response to the external environment were conserved and highly represented in σ^54^ regulons. The chlamydial σ^54^ regulon appears to share the same theme. On the basis of the findings of the current study, the regulon of σ^54^ in *Chlamydia* includes genes normally upregulated after 16 or 18 hpi during the developmental cycle and genes encoding mainly T3SS-associated and membrane proteins as well as genes which have transcripts enriched in EBs. These data support the idea of a role of σ^54^ in the preparation of the chlamydial cells for RB-to-EB conversion and in the arming for subsequent host cell infection.

## MATERIALS AND METHODS

### Overexpression and purification of recombinant CtcC protein variants.

The *ctl0728* (*ctcC*) gene was amplified via PCR from C. trachomatis (LGV2 434/Bu; GenBank accession no. CP019386.1) genomic DNA (gDNA), either the full-length open reading frame or the ATPase domain-only form (aa L142 through L386). The gene was inserted into the pTBSG vector in frame and immediately downstream of a sequence encoding an N-terminal hexahistidine tag and tobacco etch virus (TEV) protease recognition site. The E242A residue substitution was introduced via site-directed mutagenesis using a Q5 site-directed mutagenesis kit (New England Biolabs, Ipswich, MA).

After the sequence was confirmed, the vector was transformed into BL21(DE3) E. coli competent cells, which were then grown at 37°C in Terrific Broth supplemented with 100 μg/ml carbenicillin to an optical density at 600 nm (OD_600_) of approximately 0.8. Protein expression was induced with the addition of IPTG (isopropyl-1-thio-β-d-galactopyranoside) to reach a final concentration of 0.5 mM for 10 h at 17°C. Following E. coli collection by centrifugation (10,000 × *g*, 20 min), cells were resuspended in washing buffer (10 mM HEPES [pH 7.2], 5 mM EDTA, 0.1% Triton X-100) with the addition of phenylmethane sulfonyl fluoride to reach a final concentration of 1 mM and 1,000 U of Benzonase endonuclease (EDM Millipore, Burlington, MA). Cells were lysed by sonication and centrifuged for 30 min at 14,000 × *g*. The supernatant was decanted after this centrifugation, and the insoluble protein pellet was resuspended in wash buffer and centrifuged again. This wash step was repeated one additional time, for a total of three washes and centrifugations. After the final wash, the supernatant was again decanted, and the pellet was resuspended in denaturing buffer (6 M guanidine hydrochloride–phosphate-buffered saline [PBS; pH 8.0]) and rocked overnight at 4°C. The denatured cell mixture was centrifuged at 20,000 × *g* for 30 min, and then the supernatant was applied to a gravity flow HisPur cobalt resin column (Thermo Fisher, Waltham, MA). After applying the protein to the column, it was washed with three column volumes of the denaturing buffer followed by three volumes of washing buffer (8 M urea, 100 mM NaH_2_PO_4_, 10 mM imidazole at pH 8.0). A total of three column volumes of elution buffer (8 M urea, 100 mM NaH_2_PO_4_, 250 mM imidazole at pH 3.0) was used to elute the protein off the column. Dialysis of the eluted protein into refolding buffer (250 mM NaCl, 50 mM NaH_2_PO_4_, 5 mM dithiothreitol [DTT], 5% glycerol, pH 10.0) was performed with gradually lower concentrations of urea at 6, 3, 2, 1.5, 1, 0.5, 0.25, and 0 M, changing buffers at a minimum of 3 h after each concentration step. Protein concentrations were confirmed using a Bradford assay.

### *In vitro* ATPase activity assay with purified recombinant CtcC protein variants.

Quantification of the ATPase activity was performed using the protocol and reagents provided in an ADP-Glo kinase assay kit (Promega, Madison, WI). All proteins were initially suspended in the same buffer (50 mM NaH_2_PO_4_, 250 mM NaCl, 5 mM DTT, 5% glycerol at pH 10.00) and warmed to 37°C. Each were then added in a 45:45:10 volume ratio to warmed activity buffer (50 mM Tris base [pH 8.0], 100 mM KOAc, 27 mM NH_4_OAc, 8 mM MgOAc, 25 μM EDTA, 1 mM DTT) with 4 mM Mg-ATP and incubated at 37°C for 30 min. Luminescence was then measured on a plate-reading luminometer (Infinite M200 Pro; Tecan, Mannedorf, Switzerland). All experiments were performed under all conditions in triplicate in a 96-well plate format.

### Construction of *ctcC* expression constructs and *Chlamydia* transformation.

The *ctl0728* (*ctcC*) gene was amplified via PCR from C. trachomatis (LGV2 434/Bu; GenBank accession no. CP019386.1) genomic DNA, either the full-length open reading frame or the ATPase domain-only form (residues L142 through L386). The gene was inserted into the pL2-tetO overexpression plasmid using ligase-independent cloning methods at the AgeI restriction enzyme site and transformed in DH5α competent E. coli cells (49). After sequence and protein expression in E. coli cells were confirmed, vector plasmid was isolated using a Qiagen plasmid miniprep kit. The E242A substitution was introduced via site-directed mutagenesis using a Q5 site-directed mutagenesis kit (New England BioLabs, Ipswich, MA).

Purified *ctcC* expression vector plasmid was transformed into a clonal isolate of L2 C. trachomatis (LGV2 434/Bu; GenBank accession no. CP019386.1 [79]). Briefly, 15 μg of vector plasmid was mixed with 100 μl 2× CaCl_2_ buffer (20 mM Tris [pH 7.5], 100 mM CaCl_2_) and 25 μl of C. trachomatis EBs and diluted with water to reach a final volume of 200 μl. This CaCl_2_ mixture was incubated at room temperature for 30 min before being diluted into 1× CaCl_2_ buffer, added onto a confluent monolayer of L292 cells, and then centrifuged for 30 min at 550 × *g* and 20°C. Following centrifugation, the CaCl_2_ mixture was aspirated off the monolayer and replaced with RPMI media supplemented with 5% tetracycline-free fetal bovine serum (FBS), 10 μg/ml gentamicin, and 1 μg/ml cycloheximide and incubated at 37°C in 5% CO_2_. After 16 h, 1 μg/ml of ampicillin was added to the infected cells. The infection was allowed to incubate for 42 h after the initial infection, at which point the cells were lysed using water lysis and passaged to a new monolayer of cells, with media containing 1 μg/ml of ampicillin. After an additional 48 h, the cells were passaged again with 2 μg/ml of ampicillin. Two additional passages were performed 48 h after each previous passage, with new media containing 5 μg/ml of ampicillin. At that point, the infection was propagated for harvesting. The presence of the vector plasmid was confirmed by PCR, antibiotic selection, and protein expression; in addition, the plasmid was sequenced to ensure the integrity of the construct.

### Western blotting of CtcC overexpression in C. trachomatis.

L929 host cell monolayers were infected with C. trachomatis transformed with the different tet-inducible *ctcC* construct variant plasmids and incubated in RPMI media supplemented with 5% tetracycline-free FBS, 10 μg/ml gentamicin, and 1 μg/ml cycloheximide at 37°C in 5% CO_2_. Infections were induced with 10 ng/ml of anhydrotetracycline (ATc) at 16 h postinfection. At 20 h postinfection, infections were harvested by applying a 1:1 ratio of PBS (Corning, NY) and 2× Laemmli sample buffer with 2-mercaptoethanol (Bio-Rad, Hercules, CA) directly to the cell monolayer and cell scraping was done to collect the contents of the tissue culture plate well. The collected sample was used for Western blot analysis. Rabbit polyclonal primary antibody raised against full-length CtcC (Proteintech, Chicago, IL) and goat anti-major outer membrane protein (anti-MOMP) primary antibody (ViroStat, Westbrook, ME) were used to probe for the protein of interest and a loading control protein, respectively. IRDye donkey anti-rabbit 680LT and donkey anti-goat secondary 800CW antibody (Li-Cor, Lincoln, NE) were used to visualize the Western blot. Western blot analysis was performed upon plasmid transformation into *Chlamydia* as well as alongside RNA isolation and growth analyses to ensure that the observed phenotypes corresponded to the induced protein expression. Figure 2B shows a representative Western blot of the overexpression of CtcC protein variants.

### Progeny assay with induction of CtcC protein variant overexpression.

L929 host cell monolayers in 6-well tissue culture plates were infected with C. trachomatis transformed with different tet-inducible *ctcC* variant plasmids or empty pL2 vector control plasmid using RPMI supplemented with 5% tetracycline-free FBS, 10 μg/ml gentamicin, and 1 μg/ml cycloheximide. At 12 h postinfection, 10 ng/ml of ATc was added to induce overexpression of CtcC protein variants. At 30 h postinfection, the infected monolayer was lysed using a water lysis technique and passaged onto a new monolayer of host cells in a 96-well plate format to determine infectious titer. At 24 h after the passage, the 96-well plate was fixed and stained using the MicroTrack C. trachomatis culture confirmation test (Syva Co., Palo Alto, CA). Wells were then quantified for the number of inclusions, and the data were compared to the number of inclusion-forming units in the initial infection. This procedure was repeated in triplicate for each condition.

### Immunofluorescence microscopy of C. trachomatis-infected cells following expression induction of CtcC variants.

L929 cells were grown to ∼75% confluence in an 8-well ibiTreat μ-Slide (Ibidi, Martinsried, Germany) and were infected with respective C. trachomatis L2 mutants. At 12 hpi, 10 ng/ml ATc was added to the respective wells, and at 24 hpi, the wells were fixed with methanol for 10 min and then washed with Hanks’ balanced salt solution (HBSS). Cells were washed with HBSS and then stained with MicroTrack C. trachomatis culture confirmation test (Syva Co., Palo Alto, CA) and 10 nM 4′,6-diamidino-2-phenylindole (DAPI)–PBS. A final overlay of Vectashield antifade mounting medium (Burlingame, CA) was added, and the slides were immediately imaged. Cells were visualized on an Olympus IX81/3I spinning disk confocal inverted microscope at ×150 magnification and captured on an Andor Zyla 4.2 scientific complementary metal oxide semiconductor (sCMOS) camera (Belfast, Northern Ireland). Microscope and camera were operated using SlideBook 6 software (Intelligent Imaging Innovations, Denver, USA). Images are representative of at least 5 different fields/inclusions per condition. Exposure time remained consistent for all fields captured, with exposure for DAPI (DNA) at 1 s, FITC (OmpA) for 3 s, and Cy5 (cytoplasm) for 3 s. Three Z-stack images at 0.3 μm apart were taken per field imaged. Images were processed in SlideBook 6, and No Neighbors Deconvolution with a subtraction constant of 0.4 was applied to all images. Images represent an average projection over the Z-axis of the three Z-stacks for each field shown.

### RNA purification and RNA-seq.

L929 cells in a 6-well tissue culture plate with tetracycline-free RPMI media were infected with the pL2-tetO *ctcC* ATPase-only construct or the pL2-tetO vector control. The infection was induced with a final ATc concentration of 10 ng/ml at 16 hpi. A relatively small window of induction was chosen to reduce the pleiotropic effect of overactivation of σ^54^. At 20 hpi, the results of the infection was harvested as described in the previous section using TRIzol and subsequent chloroform RNA extraction. Per the procedure for RNA-sequencing analysis of intracellular bacteria described previously by Marsh, Humphrys, and Myers (80), following Turbo DNase treatment (Thermo Fisher, Waltham, MA) and purification performed using an RNeasy kit (Qiagen, Hilden, Germany), both host rRNA and chlamydial rRNA were depleted using Ribo-Zero magnetic core kits (Illumina, San Diego, CA) specific for human/mouse/rat rRNA and Gram-negative bacterial rRNA. A final purification step was performed using the RNeasy minikit (Qiagen, Hilden, Germany). rRNA depletion was assessed by running samples on a 2% agarose gel. RNA quality was assessed using Qubit quantification and TapeStation gel analysis. The RNA library was made using a NEBNext Ultra II directional RNA library prep kit for Illumina (New England BioLabs, Ipswich, MA) and was sequenced with NextSeq 550 high-output single-read 50-bp sequencing (NX-HO-SR50). Geneious Prime (Version 2019.1.1) was used for the data analysis. Reads were aligned to Chlamydia trachomatis 434/Bu genome (NC010287). Transcript levels were normalized to reads per million. Genes with an average differential expression ratio 2 standard deviations away from the mean differential expression ratio for the entire genome were considered to be significantly upregulated and were included as part of the σ^54^ regulon and listed in Table 1 or Table 2, depending on the presence or absence of a predicted σ^54^ promoter region upstream of the open reading frame. The protein roles listed in Table 1 and Table 2 were verified with data from previous studies (37, 39–41, 44, 54–57, 64, 67, 68, 71, 74, 81–104).

### Droplet digital PCR quantification of RNA transcripts with CtcC protein variant overexpression.

L292 cells in a 6-well tissue culture plate containing tetracycline-free media were infected with the different *ctcC* construct plasmids or with empty pL2 control vector, and transformed C. trachomatis cells were induced with 10 ng/ml ATc at 16 h postinfection and then harvested with 1 ml per well TRIzol. A 200-μl volume of chloroform was added to each 1-ml TRIzol cell lysate, subjected to vortex mixing, and centrifuged for 8 min at 10,000 × *g*. The aqueous layer of the TRIzol/chloroform mixture was collected in a new tube, and a 1:1 ratio of isopropanol was added before incubation was performed at –20°C for 30 min, followed by incubation at room temperature for 15 min. The precipitated nucleic acids were pelleted by centrifugation for 8 min, the supernatant was decanted, and the pellet was washed with 1 ml 70% ethanol. After another 5-min centrifugation, the pellet was air-dried and resuspended in 80 μl of RNase-free molecular-biology-grade water (Corning, NY). DNA depletion was performed by addition of 10 μl of Turbo DNase buffer and 10 μl of Turbo DNase (Thermo Fisher, Waltham, MA) to each 80 μl of sample and incubation in a 37°C water bath for 30 min. Immediately following DNA depletion, the samples were processed through the RNeasy minikit (Qiagen, Hilden, Germany) and eluted into 50 μl of RNase-free water.

cDNA was generated using a High-Capacity cDNA reverse transcription kit (Applied Biosystems, Foster City, CA) that included control reaction mixtures excluding reverse transcriptase, which was then used to assess the presence of residual gDNA contamination. Following cDNA generation, droplet digital PCR (ddPCR) was used to determine an absolute quantification for the number of transcripts present for the genes of interest. A QX200 digital droplet PCR system (Bio-Rad, Hercules, CA) with all the suggested Bio-Rad consumables, including QX200 EvaGreen Supermix, was utilized in the setup and reading of the ddPCR run. Primers for specific genes of interest used for ddPCR are listed in Table S4 in the supplemental material. Data were analyzed using QuataSoft Analysis Pro software, version 1.0 (105). The data were normalized to the quantification of *secY* transcript copies and were converted to log_2_ scale for comparisons to the number of transcripts in the empty pL2 vector control induced with ATc (33, 34).

### Miller assays.

β-Galactosidase activity was used to assess the ability of σ^54^ to activate transcription of a downstream gene (*lacZ*) from promoter regions taken from those genes found to be upregulated in the RNA-seq analysis. Promoter regions were amplified using primers listed in Table S3 and cloned into the pAC-*lacZ* vector at the BamHI restriction site upstream of the *lacZ* gene (37). The chlamydial genes encoding σ^54^ and the CtcC ATPase domain (residues 142 to 386), with and without the E242A amino acid substitution, were cloned into pRSF-Duet vector at the NcoI and NdeI restriction sites, respectively, putting the transcription of both these genes under the control of IPTG-inducible regulation. All insertions were confirmed by sequencing. E. coli cells were cotransformed with the pAC-*lacZ* plasmid containing the various promoter regions in combination with the different pRSF-Duet plasmids. Levels of IPTG-induced expression of σ^54^ and the CtcC ATPase domain were assessed via Western blotting.

10.1128/mBio.01725-20.9 TABLE S3 Primers for cloning. Download Table S3, PDF file, 0.04 MB. Copyright © 2020 Soules et al. 2020 Soules et al. This content is distributed under the terms of the Creative Commons Attribution 4.0 International license.

10.1128/mBio.01725-20.10 TABLE S4 Primers for analysis. Download Table S4, PDF file, 0.04 MB. Copyright © 2020 Soules et al. 2020 Soules et al. This content is distributed under the terms of the Creative Commons Attribution 4.0 International license.

Overnight cultures were grown in LB broth supplemented with 50 μg/ml of chloramphenicol and kanamycin (only chloramphenicol was used when just the pAC-*lacZ* vector was present). Cultures were then diluted 1:100 in fresh broth and grown at 37°C until the culture reached mid-log phase. Cultures were induced with 1 mM IPTG for 1 h before being centrifuged. Pellets were washed and resuspended in Z-buffer (0.06 M Na_2_HPO_4_, 0.04 M NaH_2_PO_4_, 0.01 M KCl, 0.001 M MgSO_4_, 0.05 M β-mercaptoethanol, pH 7.0). Permeabilization was performed with 10% volume chloroform and 5% volume 0.1% sodium dodecyl sulfate for 10 min at room temperature. A 100-μl volume of the aqueous layer of the mixture was then aliquoted in triplicate into a microtiter plate, after which 20 μl of *ortho*-nitrophenyl-β-galactoside (4 mg/ml) was added. A 50-μl volume of Na_2_CO_3_ was added to each well after 10 min to stop the reactions. OD_420_ and OD_550_ were then measured for each reaction mixture, and the OD_600_ of samples was taken prior to cell permeabilization, in order to calculate Miller units (37, 106).
