# Supplementary material for: Sigma 54-Regulated Transcription Is Associated with Membrane Reorganization and Type III Secretion Effectors during Conversion to Infectious Forms of Chlamydia trachomatis
Source: mBio. 2020 Sep 8;11(5):e01725-20. doi: 10.1128/mBio.01725-20 (PMC7482065; doi:10.1128/mBio.01725-20)
Supplement: TABLE S1 [file mBio.01725-20-st001.pdf]

Table S1. Average differential expression ratio (ATPase vs vector control).

	CtcC ATPase		E242A ATPase[a]	CtcC Full Length
Gene	RNAseq	ddPCR	ddPCR	ddPCR
ct084	4.68	28.78	1.42	1.51[b]
ct105	3.12	3.41	-1.17	1.43[b]
ct142	4.81	13.14	1.29	1.51[b]
ct229	3.67	9.11	1.18	1.47[b]
ct394	4.50	9.67	1.50	1.98[b]
ct444	4.60	22.16	1.02	1.77[b]
ct456	4.75	4.66	-1.47	2.70[c]
ct494	5.02	27.35	1.04	1.76[b]
ct576	4.83	5.10	1.09	1.31[b]
ct619	5.66	3.53	1.00	4.71[c]
ct620	6.48	27.61	1.40	5.92[b]
ct646	4.32	2.98	1.01	1.55
ct683	3.59	4.65	1.06	2.21[b]
ct711	4.36	7.19	1.15	5.42[c]
ct814	3.55	6.08	1.13	1.32[b]
ct814.1	5.24	12.47	-1.39	1.21[b]
ct847	4.03	8.00	1.79	1.66[b]
ct875	3.83	3.17	1.07	2.39
hctB	3.31	2.99	1.00	2.70
rpoA	-1.33	-1.08	1.12	1.05

[a] All genes analyzed by ddPCR for the CtcC ATPase E242A mutant had significantly different transcription levels, with the exception of the *rpoA* control (p-value <0.05).

[b] Significant differences in transcript counts for selected genes between the Full-length CtcC and the CtcC ATPase domain-only expression variants by ddPCR analysis (p-value <0.05).

[c] Significant differences in transcript counts for selected genes between the Full-length CtcC and the CtcC ATPase E242A mutant expression variants by ddPCR analysis (p-value <0.05).
