# Supplementary material for: Sigma 54-Regulated Transcription Is Associated with Membrane Reorganization and Type III Secretion Effectors during Conversion to Infectious Forms of Chlamydia trachomatis
Source: mBio. 2020 Sep 8;11(5):e01725-20. doi: 10.1128/mBio.01725-20 (PMC7482065; doi:10.1128/mBio.01725-20)
Supplement: TABLE S2 [file mBio.01725-20-st002.pdf]

Table S2. Temporal gene expression level evaluation of *ct229* and *ct646*

Gene	Average Normalized Transcript Counts			
	12 hpi	18 hpi	24 hpi	30 hpi
rpoA	338.52 +/- 18.16	323.67 +/- 10.06	350.13 +/- 17.82	357.65 +/- 20.01
ct229	29.4 +/- 17.85	31.5 +/- 11.45	36.08 +/- 11.48	116.08 +/- 36.72
ct646	82.82 +/- 9.31	207.65 +/- 17.18	224.93 +/- 10.15	282.57 +/- 79.94
