# Supplementary material for: Sigma 54-Regulated Transcription Is Associated with Membrane Reorganization and Type III Secretion Effectors during Conversion to Infectious Forms of Chlamydia trachomatis
Source: mBio. 2020 Sep 8;11(5):e01725-20. doi: 10.1128/mBio.01725-20 (PMC7482065; doi:10.1128/mBio.01725-20)
Supplement: FIG S2 [file mBio.01725-20-sf002.pdf]

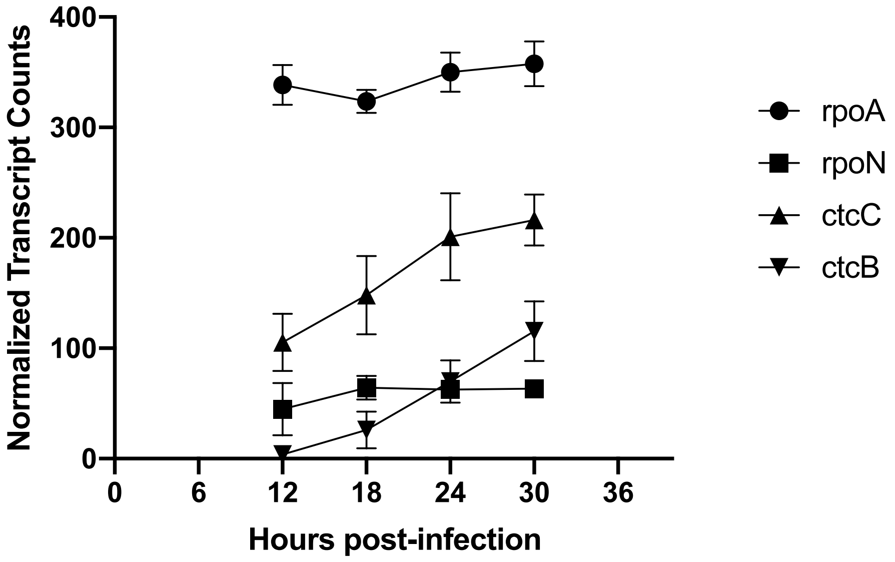

Figure S2. Temporal analysis of *ctcC, ctcB* and *rpoN* transcript levels throughout the chlamydial developmental cycle. RNA was isolated at 12, 18, 24, and 30 hpi from a wild-type L2 infection. Transcript counts were determined by ddPCR and normalized to *secY* transcript counts. *rpoA* was used as a constitutively-active control.
