# Supplementary material for: Sigma 54-Regulated Transcription Is Associated with Membrane Reorganization and Type III Secretion Effectors during Conversion to Infectious Forms of Chlamydia trachomatis
Source: mBio. 2020 Sep 8;11(5):e01725-20. doi: 10.1128/mBio.01725-20 (PMC7482065; doi:10.1128/mBio.01725-20)
Supplement: FIG S3 [file mBio.01725-20-sf003.pdf]

CT005	(0.93)	CTT **TGG** TTA-N_2-TTA **TAC** TAG
CT050	(0.93)	TAA **TGG** AGG-N_3-TGA **TAC** CTC
CT051	(0.94)	TCA **TGG** CTT-N_4-TTT **TTC** ATT
CT082	(0.94)	GCA **AGG** CCC-N_4-CCT **TGC** GTT
CT084	(0.93)	CGC **TGG** CAT-N_3-TAT **TTC** CCA
CT105	(1.00)	ATT **TGG** TAT-N_3-TAG **TGC** TTG
CT142	(0.93)	CCC **AGG** CTT-N_3-TAT **TGC** TCT
CT229	(0.94)	TTA **TGT** TAT-N_4-TTT **TGC** CAA
CT394	(0.93)	CGG **TGG** AGA-N_2-GTT **TTC** TTA
CT444	(0.93)	TGT **TTG** CTT-N_2-ATT **TGC** TAA
CT455	(0.93)	CGT **TTG** TGA-N_2-AAA **TGC** AAT
CT489	(0.94)	GTA **TGG** GTC-N_4-TTT **TGT** CAA
CT493	(0.93)	TTG **TGG** TAC-N_3-GGT **TGA** GGC
CT494	(0.93)	ACC **TGG** AGT-N_3-TAT **TTC** CAG
CT575	(0.94)	GCT **TGG** ACT-N_4-CTT **TTC** TCT
CT619	(0.93)	AAC **TCG** CAA-N_2-CCT **TGC** TCG
CT620	(0.93)	GCT **TGG** GTT-N_3-AAA **TCC** GAG
CT622	(0.94)	TGT **GGG** CTT-N_4-TAT **TGC** CTT
CT635	(0.93)	TGC **TGG** GGC-N_3-ATG **TGA** GCC
CT683	(1.00)	GAT **TGG** CAT-N_3-TTT **TGC** TCC
CT702	(0.93)	TCT **TGG** AGA-N_3-TAA **TCC** CTA
CT711	(0.93)	TTC **TGG** ATG-N_3-TCG **TGT** CAA
CT814	(1.00)	TTG **TGG** AGC-N_3-ACT **TGC** CGC
CT814.1	(0.93)	ATC **TGG** GTA-N_2-AAT **AGC** TTA
CT841	(0.93)	TAT **TGA** ATA-N_3-ATC **TGC** CTG
CT847	(1.00)	ATC **TGG** TCT-N_3-AAA **TGC** TTC
CT849.1	(0.93)	TTT **TCG** CAG-N_2-GGC **TGC** GAG
CT875	(0.93)	GGT **TGG** TTT-N_3-ATG **AGC** ATC

Figure S3. Alignment of σ^{54} promoters upstream of differentially regulated genes. Predicted σ^{54} promoters for each gene are shown along with the computational promoter prediction scores indicated in parentheses. These alignments show that the canonical TGG-N_9-TGC σ^{54} promoter sequence is conserved in these predicted promoter regions.
