# Supplementary material for: Sigma 54-Regulated Transcription Is Associated with Membrane Reorganization and Type III Secretion Effectors during Conversion to Infectious Forms of Chlamydia trachomatis
Source: mBio. 2020 Sep 8;11(5):e01725-20. doi: 10.1128/mBio.01725-20 (PMC7482065; doi:10.1128/mBio.01725-20)
Supplement: FIG S4 [file mBio.01725-20-sf004.pdf]

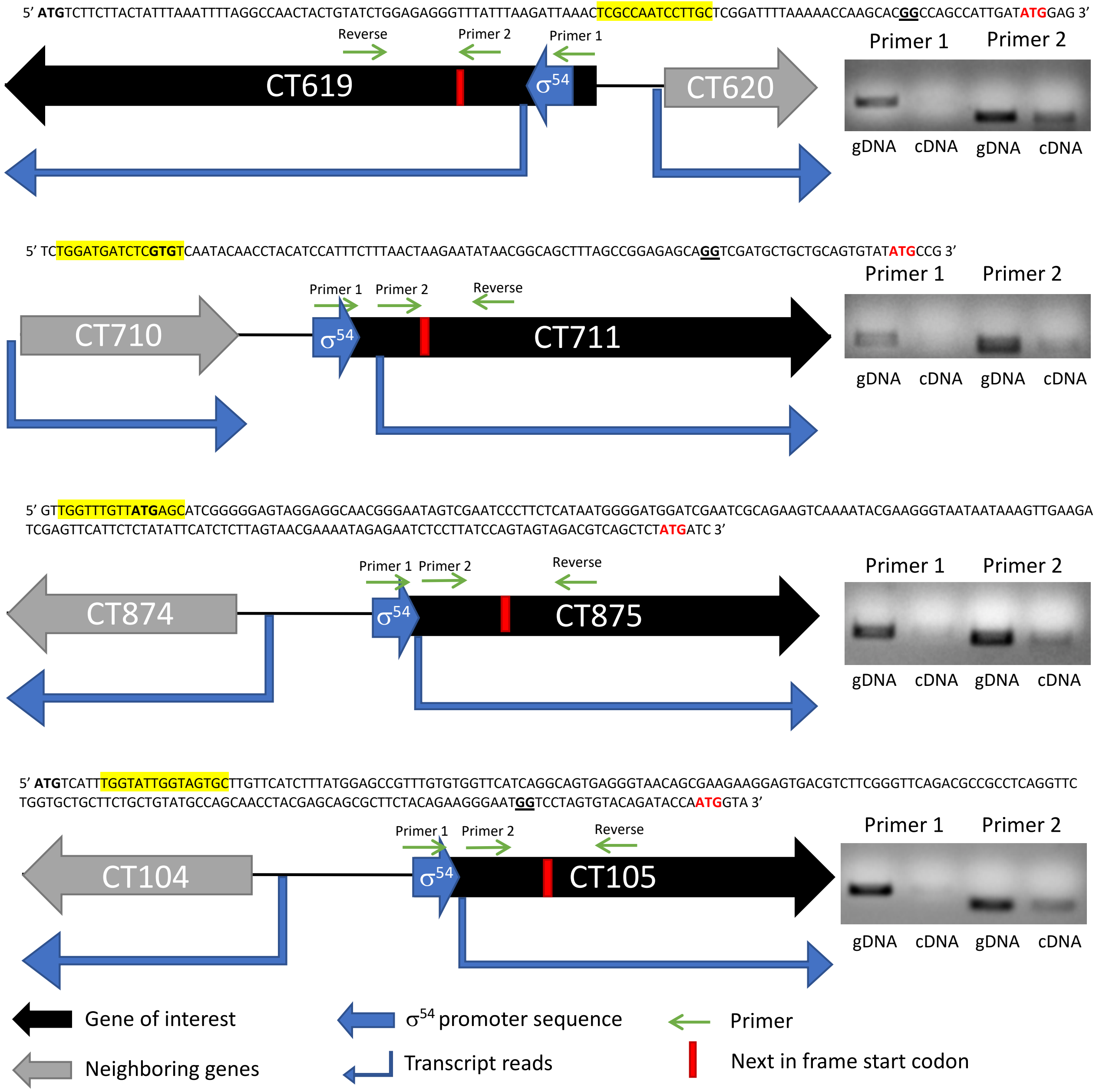

Figure S4. PCR analysis of transcriptional start site comparing the current annotated open reading frame to the transcript read alignments observed by RNAseq for four hypothetical proteins with predicted σ^{54} start sites that would affect the open reading frame of the transcribed gene. Primers were designed to sit just on either side of the transcript read alignment (blue arrow below gene), with primer 1 amplifying a region that would capture transcripts for the annotated ORF and primer 2 amplifying a region internal of the transcript alignments. The same internal reverse primer was used for PCR amplification with both primer 1 and primer 2. The approximate location of the next in frame start codon downstream from the σ^{54} promoter is represented by the red box. Above each schematic is the DNA sequence corresponding to the region including the σ^{54} promoter (highlighted in yellow), the originally annotated start codon (bold), and the next in frame ATG downstream of the σ^{54} promoter (red) with a possible ribosomal binding site within a reasonable distance (underlined, if present). To the right is the gel images showing PCR products for genomic DNA or cDNA made from isolated chlamydial RNA for each primer set. With these four genes encoding hypothetical, they show amplification only with the primer internal to the RNAseq transcript alignment supporting that the transcriptional start site is downstream from the currently annotated start codon for the gene.
