# Supplementary material for: Sigma 54-Regulated Transcription Is Associated with Membrane Reorganization and Type III Secretion Effectors during Conversion to Infectious Forms of Chlamydia trachomatis
Source: mBio. 2020 Sep 8;11(5):e01725-20. doi: 10.1128/mBio.01725-20 (PMC7482065; doi:10.1128/mBio.01725-20)
Supplement: FIG S5 [file mBio.01725-20-sf005.pdf]

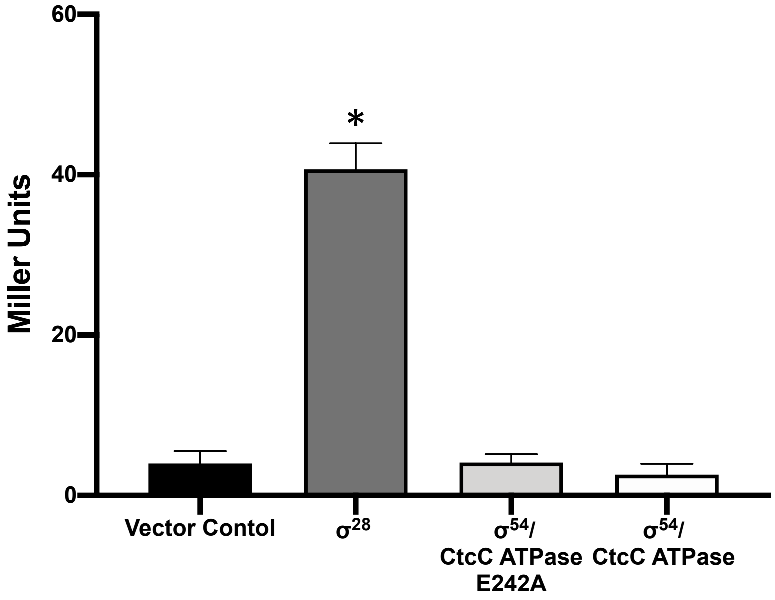

Figure S5. Beta-galactosidase assays for additional investigation of upstream regions of *hctB.* The *hctB* upstream region does not show activation of the *lacZ* gene with expression of σ^{54} and CtcC; however, when σ^{28} is present, LacZ activation is detected. * p-value < 0.05 by student t-test.
