# Supplementary material for: Sigma 54-Regulated Transcription Is Associated with Membrane Reorganization and Type III Secretion Effectors during Conversion to Infectious Forms of Chlamydia trachomatis
Source: mBio. 2020 Sep 8;11(5):e01725-20. doi: 10.1128/mBio.01725-20 (PMC7482065; doi:10.1128/mBio.01725-20)
Supplement: FIG S6 [file mBio.01725-20-sf006.pdf]

ct394 (hrcA)

CTAAAATTCTTGACCGGTGGAGACGGTTTTCTTATAATGACACCGACTTATGGAAAATAG

ct444 (omcA)

GTTTGCTTTGATTTGCTAATTACCTGTTATTAGACGATTTGTTTTAAAAAACAATTGATATAATTTTTATTTTATAATGTAATATT – N_{92} -- GTTATGAAAA

Figure S6. Upstream regions for *ct394* and *ct444* show both σ^{54} and σ^{66} promoter regions present. Promoter sequences for σ^{66} and σ^{54} are colored blue and red, respectively, with the downstream start codon for the gene underlined. Interestingly, the σ^{54} promoter lies inside of the σ^{66} promoter for *ct394.*
