# Supplementary material for: Sigma 54-Regulated Transcription Is Associated with Membrane Reorganization and Type III Secretion Effectors during Conversion to Infectious Forms of Chlamydia trachomatis
Source: mBio. 2020 Sep 8;11(5):e01725-20. doi: 10.1128/mBio.01725-20 (PMC7482065; doi:10.1128/mBio.01725-20)
Supplement: TABLE S3 [file mBio.01725-20-st003.pdf]

Table S3. Primers for cloning

Vector	Name	Restriction site	Sequence(5' --> 3')
pRSF-Deut			
	RpoN Forward	NcoI	GTTTAACTTTAATAAGGAGATATACCATGATGTTGCATCAGCATCAAACAG
	RpoN Reverse	NcoI	GGTGATGATGGTGATGGCTGCTGCCCATGTTAGATAGTATGTCGAGAATTCTCTGTGC
	CtcC ATPase Forward	NdeI	GTTAAGTATAAGAAGGAGATATACATAATGCTGATCGCCGAAAGTCCTTC
	CtcC ATPase Reverse	NdeI	CCGGCCGATATCCAATTGAGATCTGCCATATTATAAGAGAGCGAGCATAGAAGG
pACYC-lacZ			
	ct620 Promoter Forward	BamHI	CATACCCATGGGCTCTGGATCCCAAAGCTACAGCTAAACGTC
	ct620 Promoter Reverse	BamHI	GTCATCGTCATCACCGGATCCTCAACTAATGTCTTTTATAAAAAGATTACC
	ct142 Promoter Forward	BamHI	CATACCCATGGGCTCTGGATCATCCGACTCCTTACGGATAC
	ct142 Promoter Reverse	BamHI	GTCATCGTCATCACCGGATCTTAATTGTTTCCAAGTTTTTATTTTGAATAAAAAG
	ct814.1 Promoter Forward	BamHI	CATACCCATGGGCTCTGGATCGTCGGGTATCTTTACCAACAAC
	ct814.1 Promoter Reverse	BamHI	GTCATCGTCATCACCGGATCGTTTTCGATAATTATTTTCTTACTTCTTATTTAAAAAG
	ct084 Promoter Forward	BamHI	CATACCCATGGGCTCTGGATCCCCCCCATGATCCTGAATG
	ct084 Promoter Reverse	BamHI	GTCATCGTCATCACCGGATCGATTTTTTACGGCTATTTATTTTCTTTTAATTAAAAAC
	ct494 Promoter Forward	BamHI	CATACCCATGGGCTCTGGATCGGCTCTTTAGTTTACCAAGGAC
	ct494 Promoter Reverse	BamHI	GTCATCGTCATCACCGGATCGCAAACCCTAATAAATCTATTAATCGC
	ct394 Promoter Forward	BamHI	CATACCCATGGGCTCTGGATCCGGAGAGCTTCTCCGTAG
	ct394 Promoter Reverse	BamHI	GTCATCGTCATCACCGGATCAAGTCGGTGTCATTATAAGAAAACC
	hctB Promoter Forward	BamHI	CATACCCATGGGCTCTGGATCGTCGACACTAACCATTTTTTATTAAAGTTTTTC
	hctB Promoter Reverse	BamHI	GTCATCGTCATCACCGGATCGGTACCTCCCCTAATTAGACAGG
	ct229 Promoter Forward	BamHI	CATACCCATGGGCTCTGGATCAAAACCTCAGTTTTTCTGAGAGG
	ct229 Promoter Reverse	BamHI	GTCATCGTCATCACCGGATCTTATTTCCCCTAAAACTTAATGCC
	ct444 Promoter Forward	BamHI	CATACCCATGGGCTCTGGATCCCCTCCGATTCTTGTATTATATAG
	ct444 Promoter Reverse	BamHI	GTCATCGTCATCACCGGATCAACTTCCAGACTCCTTTCTAG
	ct456 Promoter Forward	BamHI	CATACCCATGGGCTCTGGATCCAAGTAATTTAGTTGTAGCATTTTTAGC
	ct456 Promoter Reverse	BamHI	GTCATCGTCATCACCGGATCAACTACAAATTAAATAAAAACAACAGCCG
	ct620 Site-Direcet Mutagenesis Promoter Bashing Forward		TAAAAACCGAGCAAGGATTGGCG
	ct620 Site-Direcet Mutagenesis Promoter Bashing Reverse		AAAACAAAGCACGGCCAGCC
pTBSG			
	Full CtcC Forward	SspI	CCGTTATCCACTTCCAATATGTCGATAGAACACATTCTTATTATTGAC
	CtcC Reverse	SspI	CCGTTATCCACTTCCAATTTATAAGAGAGCGAGCATAGAAGGAGTGA
	CtcC ATPase Forward	SspI	CCTGTACTTCCAATCCAATCTGATCGCCGAAAGTCCTTCCATG
pL2-tetO			
	Full CtcC Forward	AgeI	CTTTAAGAAGGAGATACCGGATGTCGATAGAACACATTCTTATTATTGAC
	CtcC ATPase domain Forward	AgeI	CTTTAAGAAGGAGATACCGGATGCTGATCGCCGAAAGTCCTTC
	CtcC Reverse	AgeI	CACTTCACAGGTCAACCGGTTATAAGAGAGCGAGCATAGAAGG
	CtcC E242A Site Directed Mutagenesis Forward		GCACTCTTTTACTAGATGCAATCACAGAAATTC
	CtcC E242A Site Directed Mutagenesis Reverse		CTTGGTGGGCGAGTTCAAATCTTCCTAC
