# Supplementary material for: Sigma 54-Regulated Transcription Is Associated with Membrane Reorganization and Type III Secretion Effectors during Conversion to Infectious Forms of Chlamydia trachomatis
Source: mBio. 2020 Sep 8;11(5):e01725-20. doi: 10.1128/mBio.01725-20 (PMC7482065; doi:10.1128/mBio.01725-20)
Supplement: TABLE S4 [file mBio.01725-20-st004.pdf]

**Table S4. Primers for analysis**

| Gene | Primer Position | Sequence (5' --> 3') |
|---|---|---|
| ddPCR Primers | | |
| *ct084* | Forward | TTTTTCAAACTTGTCGTGATAGCG |
| *ct084* | Reverse | AATAATGTTCAGCTTAGTTTCCCTTTTTG |
| *ct105* | Forward | GTATGCCAGCAACCTACG |
| *ct105* | Reverse | CACAAGGCTTTGCACATTAG |
| *ct142* | Forward | ATGAGTGATTCTGACAAAATTATTAATGATTG |
| *ct142* | Reverse | ACTGGTTGGGAGTCTTTCTC |
| *ct229* | Forward | CGTTGATACAATGCGACAAATG |
| *ct229* | Reverse | TTTATATTCTTCCTCAAGCTCCTC |
| *ct394* | Forward | ATGGAAAATAGAATAGAAATGTCCCAAC |
| *ct394* | Reverse | TTTCGTATCGTTGCCGAAC |
| *ct444* | Forward | GTAGTTGTTGTCGTATCGTTG |
| *ct444* | Reverse | TTGTCTGCATTTGCCGTC |
| *ct456* | Forward | CAACTTTTACATCATCAACCACTTC |
| *ct456* | Reverse | ATCGCTTGATGAGGTAGAGC |
| *ct494* | Forward | TTTTTCGATTTTTATTAAAAGGTTTTTTATCCG |
| *ct494* | Reverse | CAACTCCTTGAGCGTTGG |
| *ct576* | Forward | CCTGTTCCTACGGAAGAG |
| *ct576* | Reverse | CAAAAGCGGCTTCATCATAC |
| *ct619* | Forward | GAGTATTTCCTGGAGGAGACTG |
| *ct619* | Reverse | TTCGACGATACACCAGCTAC |
| *ct620* | Forward | ATTCTAGAAGATGCTCTTGTCTCAG |
| *ct620* | Reverse | CTAACTAGCCAGTTTTCTTGTTAAACC |
| *ct646* | Forward | GTAACCCAAAGTGCAGAAAAC |
| *ct646* | Reverse | GTCTCCTGTCCTTGTAAGG |
| *ct683* | Forward | GGAAAAGCATTTAGCGAAAGAG |
| *ct683* | Reverse | ACGTAAATAGCTGTCTCCAG |
| *ct711* | Forward | CCATTTCTTTAACTAAGAATATAACGGC |
| *ct711* | Reverse | TTTACTCGTTGTTCCCGTAG |
| *ct814* | Reverse | CTGCAAATTCTCGTGTGTG |
| *ct814.1* | Forward | ATGATTTATTTTTTTGTTTTTATGACCCCC |
| *ct814.1* | Reverse | TCGACTGACAAGTTTCATAATCTTATG |
| *ct841* | Forward | TGTTGCTTATGGTTGCGAG |
| *ct847* | Forward | AAATACTGTCATACCAGACTCTAC |
| *ct847* | Reverse | TAACAAGGGGATGTTGATTAGTTC |
| *ct875* | Forward | GAATCCCTTCTCATAATGGGG |
| *ct875* | Reverse | GGTGCTCTCGATCATAGAGC |
| *rpoA* | Forward | ATGTCGGATAGTTCACACAATTTAC |
| *rpoA* | Reverse | TTCCCAAGGTGTGCCCC |
| *secY* | Forward | TCAAGGTCTTCCTTTGGGGC |
| *secY* | Reverse | TACACAATGAATAGGGTAACTTTGC |
| TSS Position Confirmation Primers | | |
| *ct619* | Primer 1 | CTGGAGAGGGTTTATTTAAGATTAAAC |
| *ct619* | Primer 2 | GAGTATTTCCTGGAGGAGACTG |
| *ct619* | Reverse | TTCGACGATACACCAGCTAC |
| *ct711* | Primer 1 | TTAGAAAAACAATAGTTTTCTGGATG |
| *ct711* | Primer 2 | CCATTTCTTTAACTAAGAATATAACGGC |
| *ct711* | Reverse | TTTACTCGTTGTTCCCGTAG |
| *ct875* | Primer 1 | TGGGTTGGTTTGTTATGAGC |
| *ct875* | Primer 2 | GAATCCCTTCTCATAATGGGG |
| *ct875* | Reverse | GGTGCTCTCGATCATAGAGC |
| *ct105* | Primer 1 | ATGTCATTTGGTATTGGTAGTGC |
| *ct105* | Primer 2 | GTATGCCAGCAACCTACG |
| *ct105* | Reverse | CACAAGGCTTTGCACATTAG |
