# Supplementary material for: Sigma 54-Regulated Transcription Is Associated with Membrane Reorganization and Type III Secretion Effectors during Conversion to Infectious Forms of Chlamydia trachomatis
Source: mBio. 2020 Sep 8;11(5):e01725-20. doi: 10.1128/mBio.01725-20 (PMC7482065; doi:10.1128/mBio.01725-20)
Supplement: FIG S1 [file mBio.01725-20-sf001.pdf]

```
                                     Regulatory Domain            *
CtCtcC      -MSIEHILIIDDDPHILALLSEILGARNFSVSSAPGVKQAIKQISNCPFDLIISDMNMPD      59
SmDctD      MSAAPSVFLIDDDRDLRKAMQQTLELAGFTVSSFASATEALAELSADFAGIVISDIRMPG      60
StNtrC      -MQRGIVWVVDDDSSIRWVLERALAGAGLTCTTFENGNEVLAALASKTPDVLLSDIRMPG      59
PaNtrC      MSRSETVWIVDDDRSIRWVLEKALQQEGMTTVSFDSADSVIGRLGRQQPDVIISDIRMPG      60
EcAtoC      MTAINRILIVDDEDNVRRMLSTAFALQGFETHCANNGRTALHLFADIHPDVVLMDIRMPE      60
                  : ::**:  :   :.  :   .:      .   .:  :.    .::: *:.**

CtCtcC      GSGLDIIQYTKQHRPQTPILVITAFGTIQNAVEAMRFGAFNYLTKPFSPDALFTLIAKAE      119
SmDctD      MDGLALFGKVLALDPDLPMILVTGHGDIPMAVQAIQDGAYDFIAKPFAADRLVQSARRAE      120
StNtrC      MDGLALLKQIKQRHPMLPVIIMTAHSDLDAAVSAYQQGAFDYLPKPFDIDEAVALVERAI      119
PaNtrC      ASGLDLLAQIRELHPRLPVIIMTAHSDLDSAVASYQGGAFEYLPKPFDVDEAVSLVKRAN      120
EcAtoC      MDGIKALKEMRSHETRTPVILMTAYAEVETAVEALRCGAFDYVIKPFDLDELNLIVQRAL      120
             .*:  :          *::::*... :  ** : : **:::: ***  *       :*

CtCtcC      ELQALQQDNLFLQSQG--SSISHPLIAESPSMKQLLDKARRAANSSANIFVHGESGCGKE      177
SmDctD      EKRRLVMENRSLRRAAEAASEGLPLIGQTPAMERLRQTLKHIADTDVDVLVAGETGSGKE      180
StNtrC      SHYQEQQQPRNIEVNGP----TTDMIGEAPAMQDLFRIIGRLSRSSISVLINGESGTGKE      175
PaNtrC      QHAQEQQGLELPANQAR----TPEIIGEAPAMQEVFRAIGRLSHSNITVLINGESGTGKE      176
EcAtoC      QLQSMKKESRHLHQALSTSWQWGHILTNSPAMMDICKDTAKIALSQASVLISGESGTGKE      180
            .                       :: ::*:*  :     : : :.  ::: **:* ***
                                  ATPase Domain
CtCtcC      NLSFFIHKHSPRSTKPYIKVNCAAIPDTLLESEFFGHEKGAFTGATTKKVGRFELAHQGT      237
SmDctD      VVATLLHQWSRRRTGNFVALNCGALPETVIESELFGHEPGAFTGAVKKRIGRIEHASGGT      240
StNtrC      LVAHALHRHSPRAKAPFIALNMAAIPKDLIESELFGHEKGAFTGANTIRQGRFEQADGGT      235
PaNtrC      LVAHALHRHSPRAASPFIALNMAAIPKDLMESELFGHEKGAFTGAAAQRRGRFEQADGGT      236
EcAtoC      LIARAIHYNSRRAKGPFIKVNCAALPESLLESELFGHEKGAFTGAQTLRQGLFERANEGT      240
             ::  :*  * *    :: :* .*:*. ::***:**** ******   : * :*  *  **

CtCtcC      LLLDEITEIPIHLQAKLLRAIQEQEFEHIGGIKTLPVNIRFLATSNRDLEEAIETKVLRQ      297
SmDctD      LFLDEIEAMPPATQVKMLRVLEAREITPLGTNLTRPVDIRVVAAAKVDLGDPAARGDFRE      300
StNtrC      LFLDEIGDMPLDVQTRLLRVLADGQFYRVGGYAPVKVDVRIIAATHQNLERRVQEGKFRE      295
PaNtrC      LFLDEIGDMPADTQTRLLRVLADGEFYRVGGHTPVKVDVRIIAATHQNLESLVRDGKFRE      296
EcAtoC      LLLDEIGEMPLVLQAKLLRILQEREFERIGGHQTIKVDIRIIAATNRDLQAMVKEGTFRE      300
            *:****  :*   *.::** :   ::  :*      *::*.:*::: :*        :*:

CtCtcC      DLYYRLSVISLHIPPLRDRKEDILPLAHYYLEKFCKMNNKPPKTLSLEAQRNLLDYSWPG      357
SmDctD      DLYYRLNVVTLSIPPLRERRDDIPLLFSHFLARASERFGREVPAISAAMRAYLATHSWPG      360
StNtrC      DLFHRLNVIRIHLPPLRERREDIPRLARHFLQVAARELGVEAKLLHPETETALTRLAWPG      355
PaNtrC      DLFHRLNVIRIHIPRLADRREDIPALARHFLSRAAQELAVEPKLLKAETEEYLKNLGWPG      356
EcAtoC      DLFYRLNVIHLILPPLRDRREDISLLANHFLQKFSSENQRDIIDIDPMAMSLLTAWSWPG      360
            **::**.*: : :* * :*::**  *  ::*   .         :       *   .***

CtCtcC      NVRELSNVLERTVILENDPAITPSMLALL-------------------------------      386
SmDctD      NVRELSHFAERVALGVEGNLGVPAA---------------------------------AP      387
StNtrC      NVRQLENTCRWLTVMAAGQEVLIQDLPGELFEASTPDSPSHLPPDSWATLLAQWADRALR      415
PaNtrC      NVRQLENTCRWITVMASGREVHIDDLPPELLTQPQ----DSAPAANWEQALRQWADQALG      412
EcAtoC      NIRELSNVIERAVVMNSGPIIFSEDLPPQIRQPVCNAGEVK----------------TAP      404
            *:*:*.:  .  .:   .
                                  DNA Binding Domain
CtCtcC      ------------------------------------------------------------      386
SmDctD      ASSGATLPERLERYEADILKQALTAHCGDVKETLQVLGIPRKTFYDKLQRHGINRADYVE      447
StNtrC      SGHQNLLSEAQPELERTLLTTALRHTQGHKQEAARLLGWGRNTLTRKLKELGME------      469
PaNtrC      RGQSNLLDSAVPAFERIMIETALKHTAGRRRDAAVLLGWGRNTLTRKIKELGMNVDGADD      472
EcAtoC      VGERNL-KEEIKRVEKRIIMEVLEQQEGNRTRTALMLGISRRALMYKLQEYGIDPADV--      461

CtCtcC      -------------      386
SmDctD      RAGPGRPNAISKT      460
StNtrC      -------------      469
PaNtrC      EGDD---------      476
EcAtoC      -------------      461
```

Figure S1. Clustal W sequence alignment of CtcC from *Chlamydia trachomatis* (Ct) with other σ^{54}-regulatory proteins from *Sinorhizobium meliloti* (Sm), *Salmonella* Typhimurium (St), *Pseudomonas aeruginosa* (Pa), and *Escherichia coli* (Ec) shows that the overall domain structure is conserved among bacterial species. The residues involved in σ^{54} interaction are highly conserved (red box), as is the E242 residue (red arrow) responsible for polarization of the ATP molecule allowing for hydrolysis and the D54 residue that is phosphorylated by the sensor kinase protein.
